# PXDN regulated by WTAP/YTHDF1-mediated m^6^A modification activates PI3K/AKT signaling pathway through extracellular matrix remodeling to promote progression in nasopharyngeal carcinoma

**DOI:** 10.1186/s13046-025-03609-y

**Published:** 2025-12-18

**Authors:** Ying Li, Zongwei Huang, Xingwu Huang, Wanzun Lin, Qin Ding, Wankai Fu, Ronghui Chen, Jinghua Lai, Jianmin Wang, Qinying Liu, Sufang Qiu

**Affiliations:** 1 Clinical Oncology School of Fujian Medical University, Fujian Cancer Hospital, Fuzhou, Fujian China; 2 Department of Medical Oncology, Shanghai Key Laboratory of Medical Epigenetics, Institutes of Biomedical Sciences, Fudan University Shanghai Cancer Center, Fudan University, Shanghai, China; 3 Department of Oncology, Shanghai Medical College, Fudan University, Shanghai, China; 4 Fujian Key Laboratory of Advanced Technology for Cancer Screening and Early Diagnosis, Clinical Oncology School of Fujian Medical University, Fujian Cancer Hospital, Fuzhou, Fujian China; 5 Fujian Provincial Key Laboratory of Tumor Biotherapy, Clinical Oncology School of Fujian Medical University, Fujian Cancer Hospital, Fuzhou, Fujian China; 6 Fujian Key Laboratory of Translational Cancer Medicine, Fuzhou, Fujian China

**Keywords:** Nasopharyngeal carcinoma, PXDN, m^6^A, ECM remodeling, Epithelial-mesenchymal transition

## Abstract

**Background:**

Recurrence and metastasis remain the primary causes of treatment failure in nasopharyngeal carcinoma (NPC). This study aims to explore the functional role and regulatory mechanisms of peroxidasin (PXDN) in NPC progression.

**Methods:**

Weighted gene co-expression network analysis was performed to screen the module most relevant to the malignant progression of NPC from our internal cohort (Fujian cohort 1, *N* = 192), from which the PXDN was identified as the key molecule. Clinical significance of PXDN was assessed in the GEO database and NPC tissue microarrays from Fujian cohort 2 (*N* = 103). Functional experiments were used to determine the biological role of PXDN in NPC. Methylated RNA immunoprecipitation sequencing was used to identify PXDN N6-methyladenosine (m^6^A) modification site, and verified by a dual-luciferase reporter gene assay. RNA immunoprecipitation, RNA stability assay, and RNA pull-down assay were used to verify the relationship between PXDN and YTHDF1. The CRISPR/Cas9 system was used to disrupt the m⁶A motif in the PXDN gene to further validate its role. Next, transcriptome, proteomic analysis, and immunoprecipitation assay were conducted to assess downstream targets.

**Results:**

PXDN was highly expressed in NPC and associated with poor prognosis. Suppression of PXDN drastically reduced proliferation, migration, invasion, and resistance to chemoradiation in vitro, concomitant with attenuated epithelial-mesenchymal transition. Knockdown of PXDN remarkably suppressed NPC tumorigenicity and liver metastasis in vivo. Mechanistically, PXDN promotes aggressiveness by extracellular matrix remodelling to activate the ITGB1-PI3K-AKT pathway. The aberrant expression of PXDN is governed by an m^6^A-based regulatory axis, wherein YTHDF1 recognizes WTAP-mediated PXDN m^6^A methylation to enhance its RNA stability and translation. Targeted specific demethylation of PXDN m^6^A by CRISPR/Cas9 system significantly decreased the expression of PXDN.

**Conclusions:**

We reveal the crucial role of PXDN in driving NPC malignancy and the regulatory role of m^6^A methylation modification. Targeting PXDN expression or activity could be used to effectively control NPC progression.

**Supplementary Information:**

The online version contains supplementary material available at 10.1186/s13046-025-03609-y.

## Introduction

Nasopharyngeal carcinoma (NPC) represents a distinct head and neck malignancy with marked geographical prevalence and complex tumor biology [1, 2]. Although intensity-modulated radiotherapy-based strategies have enhanced locoregional control, outcomes for advanced-stage disease remain poor, with approximately 20% of patients developing recurrence or distant metastasis post-treatment [3, 4]. NPC pathogenesis involves multifactorial interactions, including Epstein-Barr virus infection, environmental triggers, and molecular alterations such as oncogene activation and tumor suppressor silencing [1]. For recurrent or metastatic NPC, therapeutic options are limited, and median overall survival stagnates at ~ 20 months, highlighting the critical need to elucidate molecular drivers of progression [5].

The epithelial-mesenchymal transition (EMT) has been implicated in promoting metastatic dissemination and therapy resistance across malignancies, including NPC [6]. Targeting EMT-related signaling pathways may thus offer a promising therapeutic avenue for NPC [7]. Recent evidence suggests that extracellular matrix (ECM) remodeling serves as both a physical scaffold and signaling platform for EMT progression [8]. Peroxidasin (PXDN), a secreted peroxidase that catalyzes collagen cross-linking in the ECM, has emerged as a potential mediator of tumor progression [9, 10]. Although PXDN overexpression correlates with poor prognosis in several carcinomas [9–12], and its prognostic value in NPC has been recently documented [13], the functional mechanisms through which PXDN promotes aggressiveness, and the regulatory basis for its overexpression in NPC, remain largely unknown.

Here, we integrate multi-omics analyses to identify PXDN as a key regulator of NPC progression. Functional and mechanistic studies reveal that PXDN promotes tumour aggressiveness via ECM-mediated activation of the ITGB1/PI3K/AKT axis. Furthermore, we delineate an N6-methyladenosine (m^6^A)-dependent regulatory mechanism in which WTAP-mediated m^6^A modification of PXDN mRNA promotes YTHDF1-dependent mRNA stabilization and consequent abnormal expression. These findings uncover novel insights into NPC pathogenesis and nominate PXDN as a potential therapeutic target in metastatic disease.

## Methods

### Study participants

This study utilized 192 biopsy-confirmed NPC tissues (Fujian cohort 1) and 19 non-cancerous nasopharyngeal mucosal samples (from chronic nasopharyngitis patients) collected at Fujian Cancer Hospital (Fujian, China; 2015–2018) for RNA sequencing. Inclusion/exclusion criteria and sequencing methodology align with our prior report [14]. Archival specimens (86 NPC biopsies; 15 normal nasopharyngeal biopsies; Fujian cohort 2) underwent retrospective immunohistochemical (IHC) analysis of PXDN expression. Two additional NPC biopsies and matched controls (collected in 2024) were subjected to methylated RNA immunoprecipitation (MeRIP) sequencing. None of the patients had undergone any form of antitumor treatment prior to biopsy. Disease-free survival (DFS; interval from treatment initiation to disease progression or all-cause death) was tracked through April 1, 2025. The study protocol (K2022-084-01) was approved by the Fujian Cancer Hospital’s Ethics Committee (Fuzhou, China), with written informed consent obtained from all participants.

### Weighted gene co-expression network analysis (WGCNA)

WGCNA was applied to transcriptomic profiles from Fujian cohort 1. Initial sample clustering (Figure S1A) identified and excluded two outliers. Using the R package “WGCNA”, we constructed co-expression networks for the remaining 190 patients with default parameters [15]. A soft-thresholding power was selected by the “pickSoftThreshold” algorithm to achieve a scale-free topology. Modules were delineated with a minimum size of 30 and merging threshold of 0.3. Trait-module associations were quantified by calculating Pearson correlation coefficients between module eigengenes and clinical variables to identify phenotype-relevant key modules.

### Cell lines and culture

Human NPC cell lines (CNE2, HK1, C666-1, HNE1) and the immortalized nasal mucosa epithelial line HNEpC were maintained at the Laboratory of Radiation Oncology and Radiobiology, Fujian Cancer Hospital (Fuzhou, China). Cells were grown in RPMI-1640 (Shanghai BasalMedia Technologies, China) supplemented with 10% fetal bovine serum (Gibco, USA) and 1% penicillin-streptomycin-amphotericin B (Biosharp, China) at 37 ℃ under 5% CO_2_.

### RNA sequencing, Western blotting assays and quantitative real-time polymerase chain reaction

The details of RNA sequencing, western blotting and quantitative real-time polymerase chain reaction (qRT-PCR) are detailed in the Supplementary Materials. Information on the antibody specifications and primer sequences used appears in Supplementary Tables 1–2. According to the expression levels of the target gene in different cell lines, HK1, CNE2, and C666-1 cells were selected for subsequent experiments.

### Small interfering RNA and lentivirus

Short hairpin RNA (shRNA) constructs targeting YTHDF1, PXDN, and WTAP were obtained from GeneChem (China), Obio Technology (China), and General Biolystems (China), respectively. Small interfering RNA (siRNA) constructs targeting PXDN and additional m^6^A regulators were designed by GenePharma (China) and General Biolystems (China). All oligonucleotide sequences are provided in Supplementary Tables 3–4.

### Co-immunoprecipitation (Co-IP) and GST pull-down assays

Co-IP assays were performed using the Smart Magnetic IP/Co-IP Kit (Tiandirenhe Biotechnology, China). For GST pull-down assays, the IPKine™ Anti-GST Magnetic IP Kit (Abbkine, USA) was employed. Briefly, lysates from 2 × 10⁷ cells were centrifuged, and supernatants were collected; 10% of each sample was reserved as “input”. The remainder was incubated overnight at 4 ℃ with rotation using an anti-PXDN antibody (5 µg, Santa Cruz, USA) or GST-tagged recombinant human PXDN protein (5 µg, Abcam, UK). Protein A/G magnetic beads or anti-GST magnetic beads were added to the mixture and rotated at 4 ℃ for 2 h. Complexes were resuspended in 1 × SDS-PAGE loading buffer, denatured at 100 ℃ for 10 min, centrifuged, and supernatants were analyzed by immunoblotting and subsequent analysis. Immunoglobulin G (IgG) served as an immunoprecipitation control.

### Cell membrane and cytoplasmic protein purification and isolation

Membrane and cytoplasmic proteins were isolated using a Cell Fractionation Kit (Beyotime Biotechnology, China). Cells were subsequently washed with phosphate-buffered saline (PBS, BasalMedia, China), then treated sequentially with cytoplasmic extraction reagent A (cytoplasmic proteins), and membrane extraction reagent B (membrane proteins). Fractions were resolved by SDS-PAGE and immunoblotted. GAPDH and Na⁺/K⁺-ATPase (ATP1A1) served as cytoplasmic and membrane loading controls, respectively.

### Collagen gel contraction assay

A suspension of 2 × 10^6^ cells was combined with 200 µL of collagen I (Ibidi, Germany) and transferred to a 24-well plate. Following a 2 h incubation to facilitate gel polymerization, the gel was detached from the plate surfaces using a pipette tip. After 48 h of additional incubation, images of gel shrinkage were captured, and the surface area was quantified with ImageJ software.

### Cell adhesion assay

Cell adhesion was assessed using the CytoSelect™ 48-Well Cell Adhesion Assay, ECM Array (Cell Biolabs, USA). Serum-starved cells were seeded onto an ECM-coated 48-well plate at 1 × 10^5^ cells per well and allowed to adhere for 1 h. Adherent cells were then stained per the manufacturer’s protocol and quantified spectrophotometrically at 560 nm using an Infinite^®^ 200 Pro instrument (Tecan).

### RNA Immunoprecipitation (RIP) assay

RIP assays were conducted using a commercial kit (Bersinbio, China) according to the manufacturer’s protocol. Briefly, cells were lysed in RIP buffer containing protease and RNase inhibitors. Lysates were incubated overnight at 4 ℃ with protein A/G magnetic beads conjugated to anti-IgG, anti-YTHDFs or anti-WTAP antibodies. After washing, RNA-protein complexes were digested with proteinase K. RNA was extracted via phenol-chloroform purification and quantified by qRT-PCR.

### m^6^A site prediction and MeRIP assay

Potential m⁶A modification sites were predicted using the online tool SRAMP (www.cuilab.cn/sramp) [16]. MeRIP assays employed the GenSeq^®^ m⁶A MeRIP Kit (GenSeq Inc., China). Fragmented total RNA (70 ℃, 6 min) was divided: 10% served as “input”, while the remainder was incubated with anti-m⁶A or anti-IgG antibody-bound A/G magnetic beads (1 h, room temperature). After rotation (4 ℃, 1 h) and three washes, RNA was purified for qRT-PCR analysis.

### RNA pull-down assay

RNA pull-down assay was performed by Cloudseq Biotech Inc. (Shanghai, China) according to the published procedure. Briefly, biotin-labeled probes targeting m⁶A peaks of PXDN (**Supplementary Table 5**) were incubated with cytosolic extracts (37 ℃, 30 min, rotation). Streptavidin magnetic beads captured RNA-protein complexes, followed by elution and western blotting to identify interacting proteins.

### RNA stability assay

Cells treated with 5 µg/mL actinomycin D (MedChemExpress, USA) to inhibit transcription were harvested at pre-designated intervals for qPCR analysis to calculate RNA degradation rate.

### ligated intoBbsI(New England Biolabs)Dual-luciferase reporter assay

Luciferase reporter plasmids (psiCheck-2) harbouring either wild-type (WT) or mutant (mutant base substitution: A→C; Mut) sequences within the PXDN 3’ untranslated region (UTR) were constructed (Hanbio, China). Cells were transfected with WT or Mut plasmids using Lipofectamine 3000 (Invitrogen, USA). Luciferase activity was quantified using the Dual-Luciferase Reporter Assay Kit (Beyotime Biotechnology, China), following the manufacturer’s protocol. Relative luciferase activity was calculated as Renilla luciferase activity normalized to firefly luciferase activity. Plasmid sequences are detailed in Supplementary Table 6.

### CRISPR/Cas9 genome editing

Single-guide RNA (sgRNA) target sequences, designed to disrupt the m^6^A motif of PXDN, were identified using the CRISPR DESIGN tool (http://CRISPR.mit.edu) and synthesized (Hanbio, China). Oligonucleotide duplexes corresponding to the selected sgRNA were ligated into *BbsI* (New England Biolabs)-cut PHB-pSpCas9(BB)-2 A-Puro(PX459) to generate the Cas9-sgRNA expression plasmid. A donor vector containing homology arms flanking the target site was also constructed. NPC cells were co-transfected with the Cas9-sgRNA plasmid and donor vector using Lipofectamine 3000, followed by selection with puromycin. The sgRNA sequences are provided in Supplementary Table 7.

### In vivo nude mouse models

Female BALB/c nude mice (4 weeks old) were procured from Hangyi Biotech (China) and housed in the Animal Experiment Center of Fujian Cancer Hospital. All animal procedures were approved by the Laboratory Animal Ethics Committee of Fujian Medical University. Experiments, including animal care, injections, euthanasia, dissection, and tissue collection, were performed in compliance with institutional guidelines.

For the subcutaneous xenograft model, mice were randomized into two groups (*n* = 6 per group) and injected subcutaneously with HK1 cells (5 × 10⁶ cells in 100 µL PBS) transfected with either sh-PXDN or a non-targeting control (sh-NC). Tumor dimensions (length [L] and width [W]) were measured every three days using Vernier calipers, and tumor volume was calculated as (W² × L)/2. After 21 days post-injection, mice were euthanized, and tumors were excised, weighed, fixed, and paraffin-embedded. IHC staining was performed using antibodies against Ki67 and PXDN. Additionally, Masson’s trichrome and Movat-pentachrome staining were conducted following standard protocols (Solarbio, China).

For the metastasis assay, mice (*n* = 6 per group) were intravenously injected with 1 × 10⁶ sh-PXDN- or sh-NC-transfected HK1 cells in 100 µL PBS via the tail vein. After 8 weeks, lungs and livers were harvested, paraffin-embedded, sectioned, and subjected to hematoxylin and eosin (H&E) staining for metastatic lesion analysis.

### Statistical analysis

Unless specified otherwise, continuous variables were compared between two groups using unpaired Student’s *t*-test or Mann-Whitney *U* test, and among multiple groups via one-way ANOVA. Categorical variables were assessed with χ² or Fisher’s exact test. Relationship strength was evaluated by Pearson’s correlation analysis. Survival curves, generated using the Kaplan-Meier method, were compared via log-rank test. Candidate prognostic genes for DFS in NPC were identified through univariate Cox regression. The univariate and multivariate Cox regression analyses were used to identify independent prognostic factors. Prognostic accuracy was validated by plotting the area under receiver operating characteristic curves. Overlapping genes were visualized using the “venn” R package, and Kyoto Encyclopedia of Genes and Genomes (KEGG) enrichment analysis (|fold change| >1, adjusted *P* < 0.05) was performed with “clusterProfiler”.

All analyses and visualizations employed GraphPad Prism (v8.0.2, GraphPad Inc., USA), ImageJ (v1.52a, NIH, USA), and R (v4.3.3, https://www.r-project.org/). Data are expressed as mean ± SD. Statistical significance was defined as *P* < 0.05. Experiments were repeated in triplicate. Additional methodologies are detailed in the Supplementary Materials.

## Result

### PXDN is a key driver of malignant progression in NPC

**Fig. 1:**
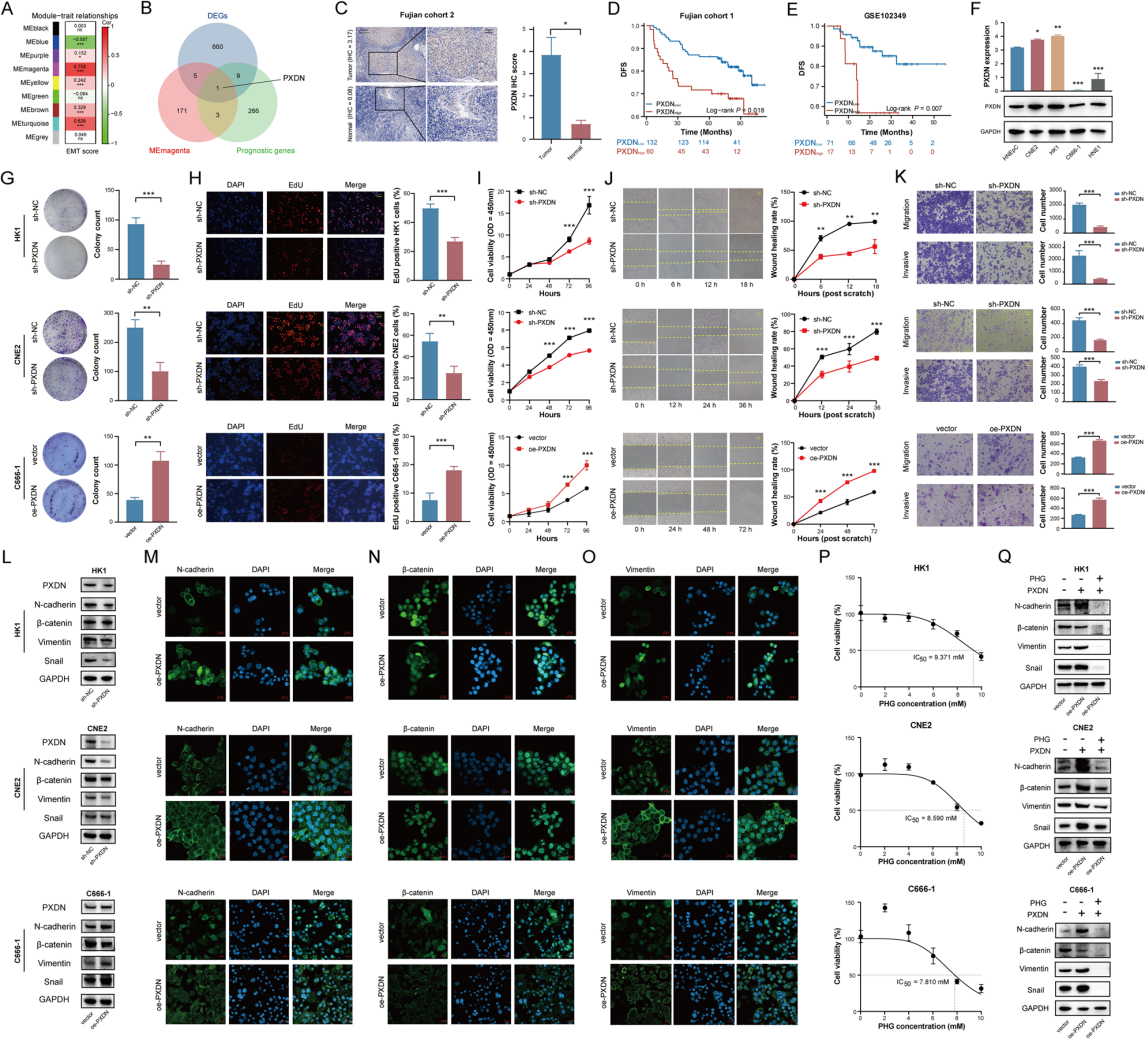
PXDN promotes aggressive phenotypes in NPC. **A** WGCNA identifying an EMT-associated module (magenta) in Fujian cohort 1. **B** Intersection of DEGs, DFS-associated genes, and magenta module genes in Fujian cohort 1. **C** PXDN protein expression in NPC versus normal nasopharyngeal tissues (Fujian cohort 2) by IHC. Left: Representative images (Scale bars: 125 μm [overview], 40 μm [inset]). Right: Quantification of staining intensity. **D**, **E** Kaplan-Meier curves of DFS in NPC patients stratified by PXDN expression (cut-off value: 8.7) in Fujian cohort 1 and GSE102349. **F** Basal expression levels of PXDN in HNEpC and NPC cells determined by RNA-sequencing and western blotting. The P values all relative to HNEpC cells. **G-I** Cell proliferation assays by colony formation, EdU, and CCK-8 assays. Scale bar: 100 μm. **J** Wound healing assays. Scale bar: 100 μm. **K** Migration and invasion assays. Scale bar: 100 μm. **L** Western blotting analysis of EMT markers following PXDN knockdown or overexpression. **M-O** Representative immunofluorescence staining showing expression of EMT markers in PXDN-overexpressing (oe-PXDN) versus control (vector) cells. Scale bar: 20 μm. **P** Dose-response curves for PHG treatment (0–10 mM, 48 h) determining IC_50_ values. **Q** PHG-induced reversal of EMT marker expression (9 mM for HK1 and CNE2, 8 mM for C666-1; 48 h) by western blotting. *, *P* < 0.05, **, *P* < 0.01, ***, *P* < 0.001. *CCK*-8 Cell counting kit-8, *Cor* Correlation, *DEGs* Differentially expressed genes, *DFS* Disease-free survival, *EMT* Epithelial-mesenchymal transition, *IC*_50_ Half maximal inhibitory concentration, *IHC* Immunohistochemistry, *ME* Module eigengene, *NPC* Nasopharyngeal carcinoma, *PHG* Phloroglucinol, *WGCNA* Weighted gene co-expression network analysis

To characterize regulators of NPC progression (Figure S2), WGCNA was performed to screen the most relevant module associated with EMT of samples from Fujian cohort 1. After removing two outliers, an optimal soft-thresholding power of 5 (R² = 0.9) was determined (Figure S1B-C), yielding nine co-expression modules following module merging (Figure S1D). The magenta module exhibited the strongest EMT correlation (Cor = 0.758, *P* < 0.001; Fig. 1A), suggesting it was a hub module related to malignant progression. Integrating differentially expressed genes (DEGs; tumor vs. normal tissue), DFS-associated prognostic genes from multiple cohorts (Figure S1E-F), and hub genes from the magenta module, we identified PXDN as a key regulator (Fig. 1B). Specifically, PXDN was upregulated in NPC tissues (Figure S1G-I; validated in Fujian cohort 2, Fig. 1C) and served as a robust diagnostic marker (Figure S1J). High PXDN expression predicted increased progression risk (Fig. 1D-E) and independently correlated with poor DFS in multivariate analysis (Figure S1K). These results indicate PXDN as a critical driver of NPC progression. The clinical relevance of PXDN was further substantiated across independent head and neck cancer cohorts, where its high expression consistently predicted poorer patient outcomes (Figure S3).

### PXDN promotes NPC proliferation, migration, invasion and EMT

To define the functional role of PXDN in NPC, we began by profiling its expression across NPC cell lines. RNA sequencing and immunoblotting revealed that PXDN expression was highest in HK1 and CNE2 cells and lowest in C666-1 cells, relative to normal HNEpC controls (Fig. 1F). Both transient siRNA transfection and stable lentiviral shRNA-mediated knockdown/overexpression efficiently suppressed/promoted PXDN expression at the mRNA and protein level (Figure S4). Functional assays demonstrated that PXDN knockdown markedly impaired the proliferative, migratory, and invasive capacities of HK1 and CNE2 cells. Conversely, PXDN overexpression significantly enhanced these oncogenic phenotypes in C666-1 cells (Fig. 1G-K). Given PXDN’s established link to EMT—a process enabling metastatic dissemination—we assessed the changes in PXDN-mediated EMT markers. Knockdown of PXDN in HK1 cells increased β-catenin levels and decreased the expression of mesenchymal markers (N-cadherin, Vimentin, and Snail). Conversely, PXDN overexpression in C666-1 cells decreased β-catenin expression and enhanced mesenchymal marker expression. A similar reduction in these mesenchymal markers was observed in CNE2 cells upon PXDN knockdown, although no significant change in β-catenin was observed in this cell line (Fig. 1L). Immunofluorescence validated these shifts (Fig. 1M-O). Conversely, phloroglucinol (PHG), a PXDN inhibitor, reversed the EMT marker expression induced by PXDN overexpression (Fig. 1P-Q). These findings establish PXDN as a regulator of EMT-driven NPC metastasis.

### PXDN facilitates radioresistance and chemoresistance in NPC cells

**Fig. 2:**
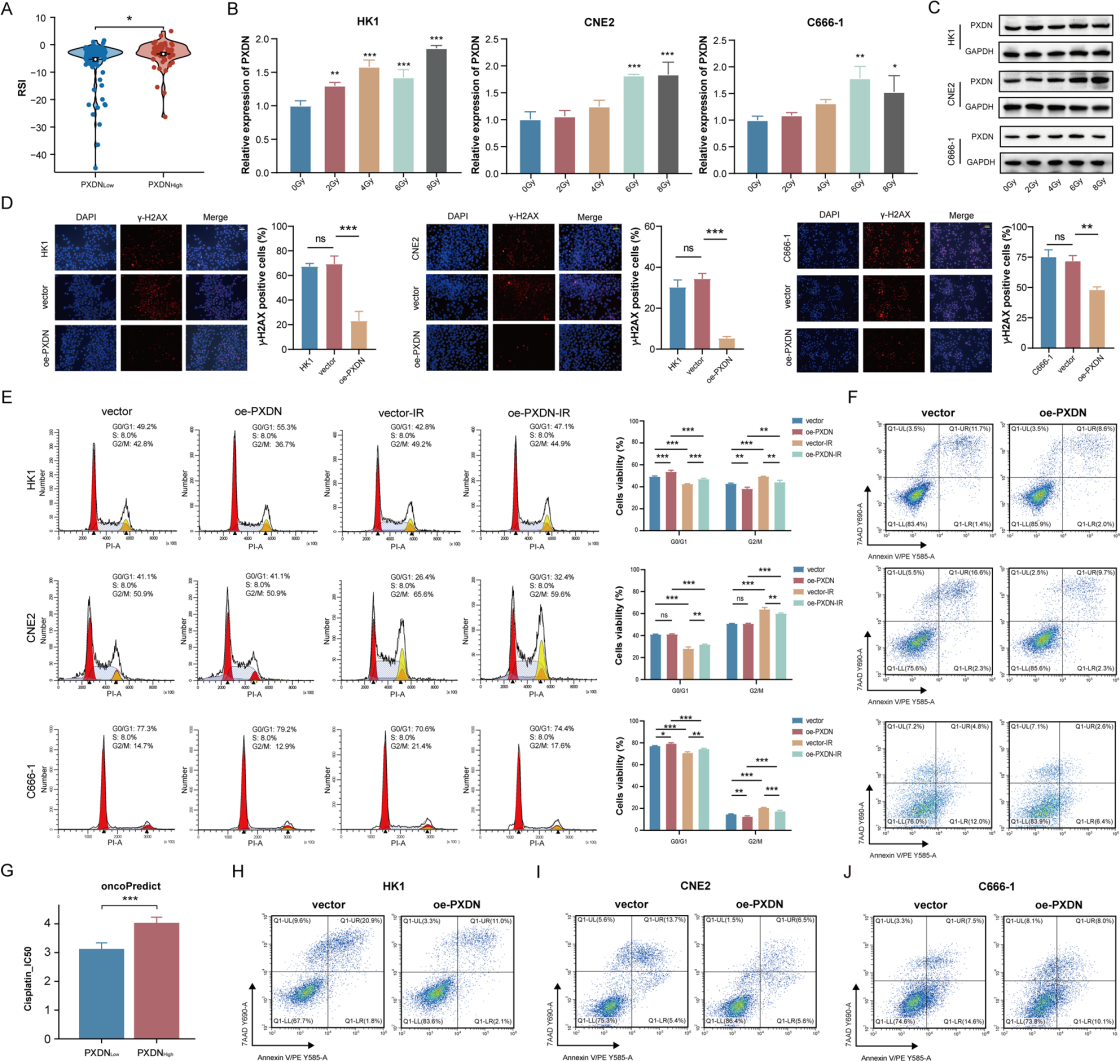
PXDN promotes radioresistance and chemoresistance in NPC. **A** The RSI in NPC patients stratified by PXDN expression in Fujian cohort 1. **B**, **C** PXDN expression following irradiation assessed by qRT-PCR and western blotting. (**D**) γ-H2AX foci formation in NPC cells with or without PXDN overexpression following irradiation. Scale bar: 50 μm. (**E**) Representative images (left) and quantification (right) of cell cycle distribution in the irradiated NPC cells. **F** Apoptosis analysis in the irradiated NPC cells. **G** Computational prediction of cisplatin IC_50_ correlation with PXDN expression (oncoPredict algorithm; Fujian cohort 1). **H**-**J** Cisplatin-induced apoptosis (10 µg/mL for HK1 and C666-1, 7 µg/mL for CNE2; 48 h) in NPC cells with or without PXDN modulation. *, *P* < 0.05, **, *P* < 0.01, ***, *P* < 0.001. *DDP* Cisplatin, *IC*_50_ Half maximal inhibitory concentration, *IR* Irradiation, *NPC* Nasopharyngeal carcinoma, *qRT*-*PCR* Quantitative reverse transcription polymerase chain reaction, *RSI* Radiation sensitivity index

Given radiotherapy as the cornerstone of NPC treatment, we first examined PXDN’s clinical relevance through radiation sensitivity index (RSI) analysis. Patients with higher PXDN expression had higher RSI scores, indicating lower sensitivity to irradiation **(**Fig. 2A). Next, we found irradiation exposure upregulated PXDN expression in a dose-dependent manner (Fig. 2B-C). We treated HK1, CNE2 and C666-1 cells with 2 Gy, 6 Gy, and 6 Gy, respectively. PXDN overexpression enhanced radioresistance, as evidenced by clonogenic survival and CCK-8 assays (**Figure S5A-F**). PXDN overexpression attenuated irradiation-induced DNA damage (reduced γ-H2AX foci at 6 h post-irradiation; Fig. 2D), promoted G2/M arrest (Fig. 2E), and suppressed apoptosis (Fig. 2F).

Cisplatin is a commonly used chemotherapy agent that, when used in combination with radiation therapy, can significantly improve the prognosis of NPC patients. We extended these findings to cisplatin resistance. The “oncoPredict” algorithm revealed higher half maximal inhibitory concentration (IC_50_) of cisplatin in PXDN-high tumors (Fig. 2G), suggesting intrinsic resistance. This was experimentally validated in NPC cells, where PXDN overexpression increased cisplatin (Noxin-Cisplatin Injection, China) IC_50_ values (Figure S5G-H). Using specific IC_50_ concentrations (10 µg/mL for HK1 and C666-1, 7 µg/mL for CNE2; Figure S5G), we demonstrated that PXDN overexpression conferred resistance by both impairing cisplatin’s anti-proliferative effects (Figure S5I-K) and suppressing drug-induced apoptosis (Fig. 2H-J).

### PXDN exhibits a positive correlation with the PI3K/AKT signaling pathway

**Fig. 3:**
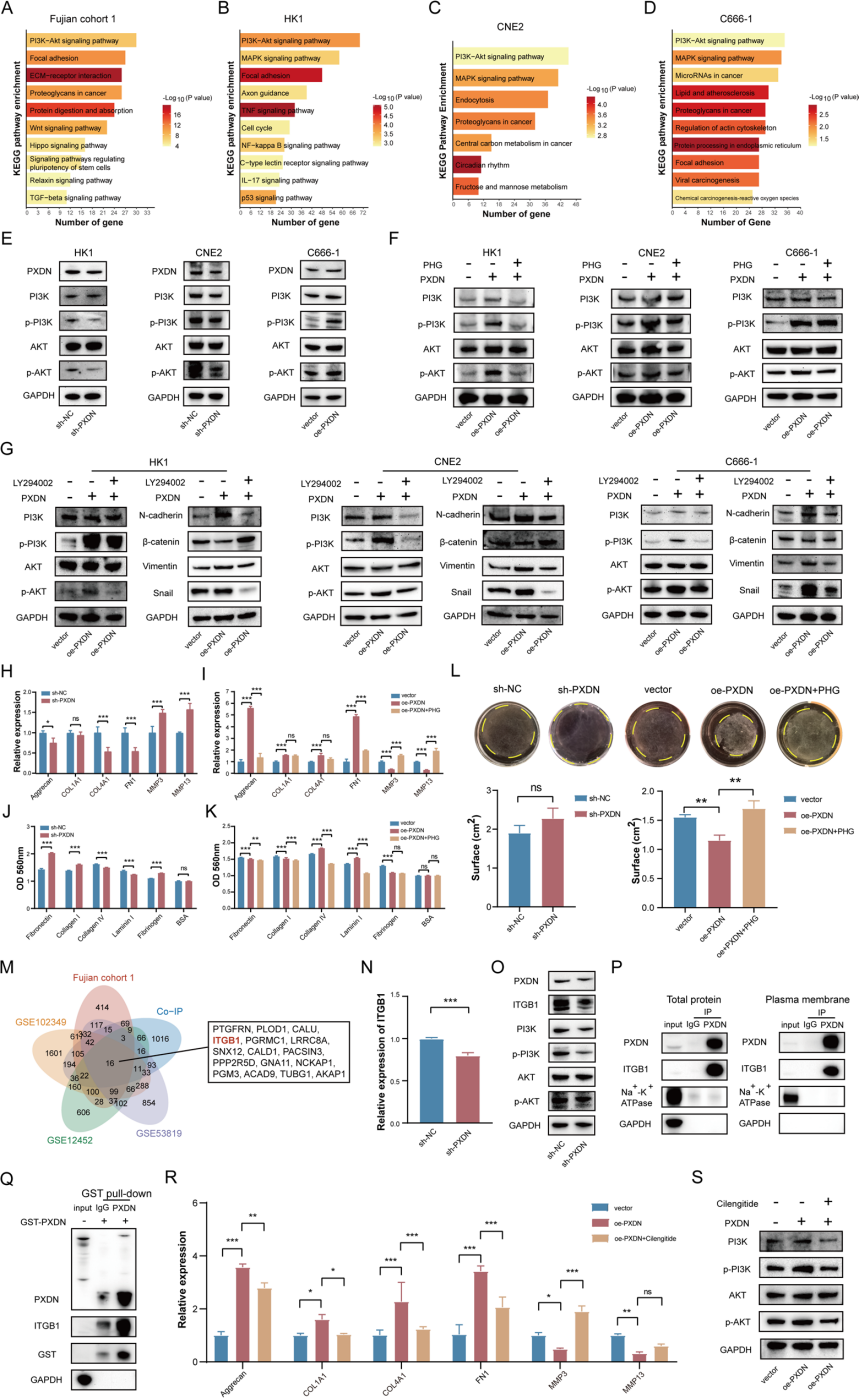
PXDN promotes NPC progression through ECM remodeling to induce the PI3K/AKT pathway. **A** The top 10 enrichment results of KEGG analysis in high- vs. low-PXDN-expressing NPC (Fujian cohort 1). **B** The top 10 enrichment results of KEGG analysis in HK1 cells with PXDN-knockdown vs. control. **C** The top enrichment results of KEGG analysis in CNE2 cells with PXDN-knockdown vs. control. **D** The top 10 enrichment results of KEGG analysis in C666-1 cells with PXDN-overexpression vs. control. **E** Western blotting analysis of PI3K/AKT signaling pathway in NPC cells. **F**, **G** Western blotting analysis of PI3K/AKT signaling pathway in PXDNoverexpressing NPC cells following PHG and LY294002 treatment. **H**, **I** qRT-PCR analysis of ECM-related genes in PXDN-knockdown or PXDN-overexpressing HK1 cells with or without PHG. **J**, **K** Cell adhesion assays in PXDN-knockdown or PXDN-overexpressing HK1 cells with or without PHG. **L** Representative image (up) and quantification (down) of collagen gel contraction assay with HK1 cells. Yellow circles denote gel boundaries. **M** Overlap of PXDN-interacting proteins (Co-IP vs. IgG) and PXDN-correlated genes (cohorts: *R* > 0.3, *P* < 0.05). **N**, **O** ITGB1 expression in PXDN-knockdown HK1 cells by qRT-PCR and western blotting. **P**, **Q** PXDN-ITGB1 interaction validated by Co-IP and GST pull-down. **R**, **S** ECM gene expression and PI3K/AKT signaling in PXDN-overexpressing HK1 cells treated with cilengitide. *, *P* < 0.05, **, *P* < 0.01, ***, *P* < 0.001. *Co*-*IP* Co-immunoprecipitation, *ECM* Extracellular matrix, *NPC* Nasopharyngeal carcinoma, *PHG* Phloroglucinol, *qRT*-*PCR* Quantitative real-time polymerase chain reaction

To further explore the mechanism by which PXDN regulates the progression of NPC, we performed functional enrichment analysis on PXDN high/low expression groups (Fujian cohort 1). KEGG pathway analysis revealed significant enrichment of DEGs in phosphatidylinositol 3-kinase/protein kinase B (PI3K/AKT) signaling, focal adhesion, and ECM-receptor interactions (Fig. 3A). Consistent with this, transcriptomic analysis of PXDN-knockdown or overexpressing NPC cells substantiated a predominant role for the PI3K/AKT signaling pathway in NPC cell lines (Fig. 3B-D). The PI3K/AKT signaling pathway is one of the most commonly activated pathways in human cancers. At the protein level, PXDN knockdown substantially reduced PI3K and AKT phosphorylation (Fig. 3E), while the PHG reversed PXDN-overexpression-induced AKT activation (Fig. 3F). Functional interrogation using the PI3K inhibitor LY294002 demonstrated that it rescued the enhanced proliferative, migratory, and invasive phenotypes conferred by PXDN overexpression (Figure S6). Furthermore, LY294002 treatment effectively blocked PXDN-mediated EMT (Fig. 3G). Taken together, our findings demonstrate that PXDN drives EMT and tumor progression in NPC through activation of the PI3K/AKT signaling axis.

### PXDN drives NPC progression via ECM remodeling to induce the PI3K/AKT pathway

Proteomic analysis of HK1 cells with PXDN knockdown identified 578 significantly differentially expressed proteins (Figure S7A-C). KEGG enrichment analysis of differentially expressed proteins showed that PXDN knockdown could affect ECM-receptor interaction pathway, consistent with our findings above (Figure S7D). Given the established association between ECM pathways and tumor metastasis, we hypothesized that PXDN may promote the development of NPC by facilitating ECM signal transduction. To verify the outcomes, we examined the mRNA levels of common ECM molecules and found that PXDN knockdown or overexpression would affect the expressions of aggrecan, COL1A1, COL4A1, FN1, MMP3 and MMP13 (Fig. 3H-I). Cell-matrix adhesion assays revealed that PXDN knockdown reduced adhesion to collagen IV and laminin I but enhanced adhesion to fibronectin, collagen I, and fibrinogen; PXDN overexpression reversed these effects (Fig. 3J). PHG counteracted PXDN-induced adhesion changes (Fig. 3K). Collagen gel contraction assays further demonstrated that PXDN overexpression amplified contractility, whereas knockdown or PHG treatment suppressed it (Fig. 3L). Overall, these results suggest that PXDN mediates alterations in the ECM, which may establish a favorable microenvironment to facilitate NPC metastasis.

To identify PXDN-interacting proteins, we performed Co-IP assays in HK1 cells followed by mass spectrometry. IP/mass spectrometry analysis combined with genes displaying significant correlations with PXDN levels (*R* >0.3 and *P* < 0.05) in the Fujian cohort 1, GSE53819, GSE12452 and GSE102349 datasets identified 16 potential PXDN-interacting proteins (Fig. 3M). Among these, we focused on ITGB1-a transmembrane integrin that mediates cell-ECM adhesion and regulates migration, survival, and proliferation through intracellular signaling [17]. PXDN knockdown suppressed ITGB1 expression at both mRNA and protein levels (Fig. 3N-O). Co-IP and GST pull-down assays confirmed direct PXDN-ITGB1 binding (Fig. 3P-Q). The integrin inhibitor cilengitide attenuated ECM-related molecule expression and abolished PXDN-driven PI3K/AKT activation (Fig. 3R-S).

### PXDN enhances proliferation and metastasis of NPC cells in vivo

**Fig. 4:**
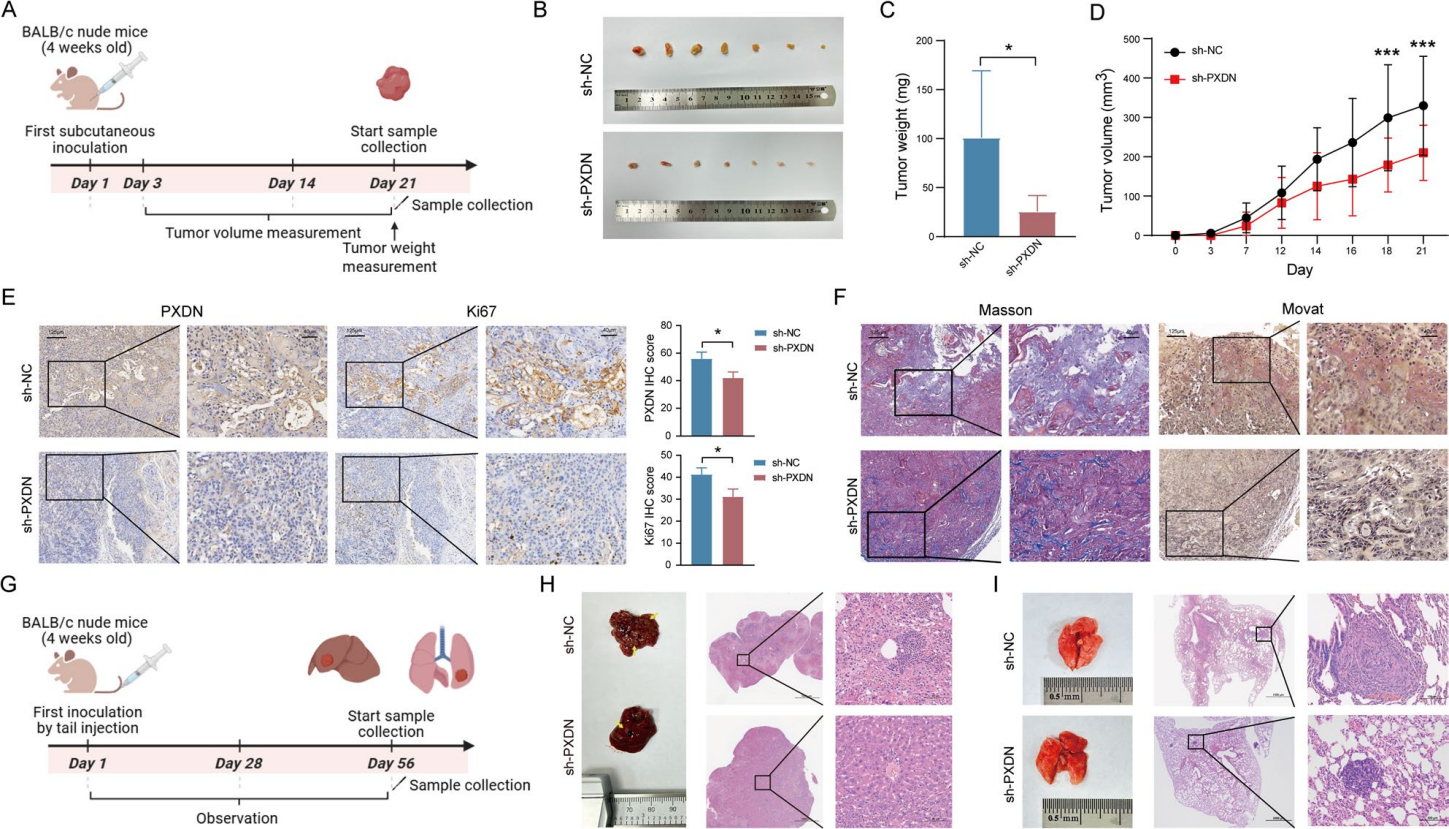
PXDN knockdown suppresses tumorigenesis and metastasis in vivo. **A** Experimental timeline of subcutaneous xenograft model (*n* = 6 mice per group). **B** Images of excised subcutaneous tumors from control (sh-NC) and PXDN-knockdown (sh-PXDN) groups at endpoint. **C**, **D** Weights and growth curves of subcutaneous tumors. **E** Representative images and quantification of IHC staining of the subcutaneous tumor with PXDN and Ki67. Scale bars: 125 μm (overview), 40 μm (inset). **F** Representative images of tumor stromal composition by Masson’s trichrome (collagen, blue) and Movat pentachrome staining (collagen, brown) (*n* = 3 biologically independent samples). Scale bars: 125 μm (overview), 40 μm (inset). **G** Experimental timeline of tail vein injection metastasis assay (*n* = 6 mice/group). **H**, **I** Hematoxylin and eosin (H&E)-stained liver and lung sections showing metastatic nodules. Scale bars: 1000 μm (overview), 80 μm (inset). *, *P* < 0.05, **, *P* < 0.01, ***, *P* < 0.001. *IHC* Immunohistochemistry

To assess the tumor-promoting role of PXDN in NPC in vivo, we employed subcutaneous xenograft and lung metastasis mouse models (Fig. 4A, G). HK1 cells with PXDN knockdown or control vectors were implanted subcutaneously into nude mice. PXDN suppression markedly reduced xenograft growth (Fig. 4B). Tumors harvested at 3 weeks post-inoculation showed significantly smaller volumes and weights in the PXDN-knockdown group versus controls (Fig. 4C-D). Immunohistochemistry confirmed PXDN downregulation in sh-PXDN tumors, which also exhibited fewer Ki67-positive cells (Fig. 4E), indicating PXDN-mediated proliferation in vivo. Masson’s trichrome staining and Movat pentachrome staining revealed reduced collagen deposition (a major ECM component) in the knockdown group and a more disordered ECM structure in the control group (Fig. 4F). In metastasis models, PXDN knockdown decreased liver nodule formation, but had no significant effect on lung metastasis in the metastasis mouse model (Fig. 4H-I).

### PXDN is epigenetically regulated by m^6^A modification

**Fig. 5:**
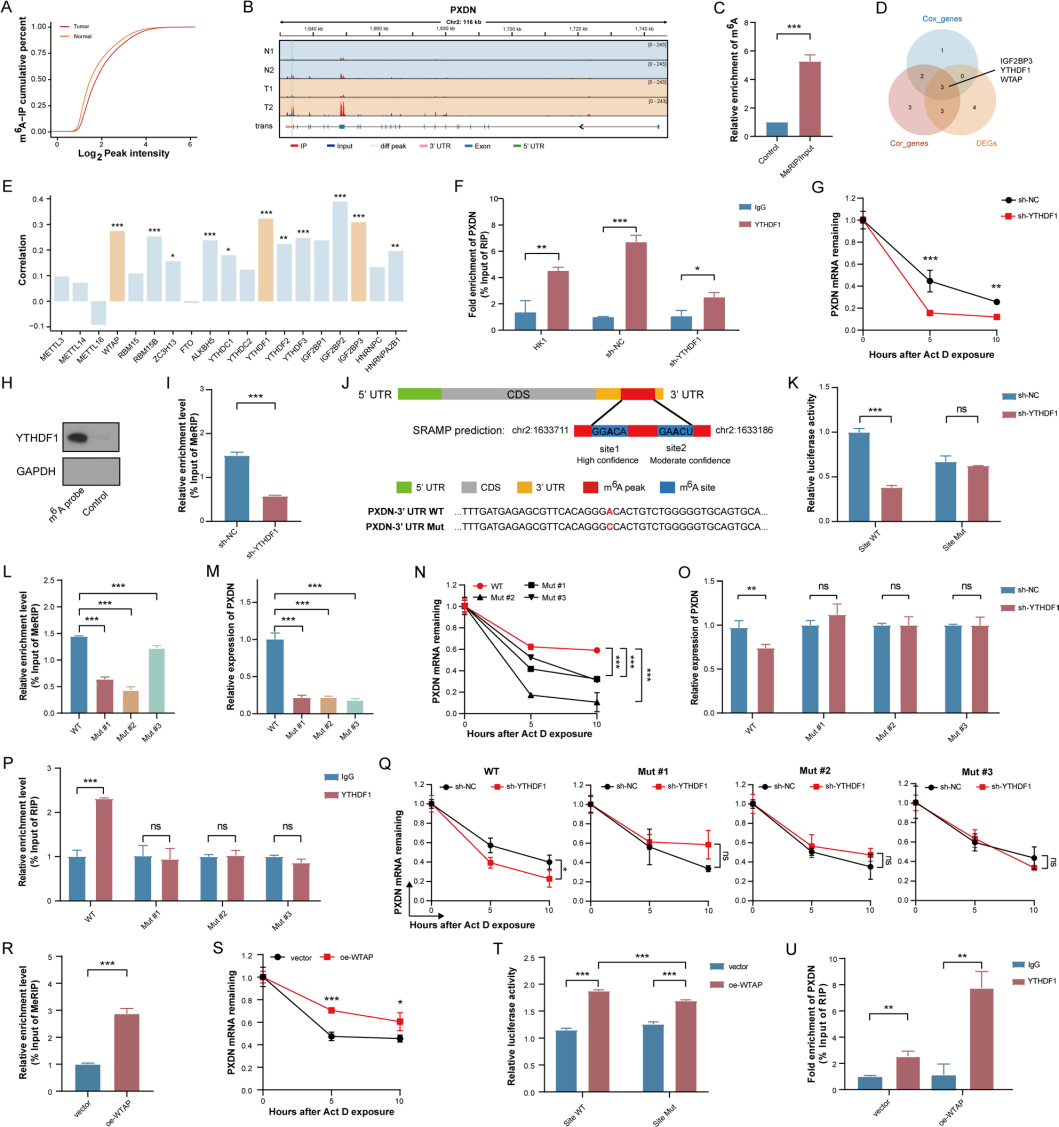
m^6^A-dependent regulation of PXDN in NPC. **A** Cumulative distribution of m^6^A methylation in NPC and normal nasopharyngeal tissues. **B** m^6^A peak distribution across PXDN transcript (Light green: NPC-specific peaks). **C** MeRIP-qPCR quantification of m^6^A enrichment at PXDN 3’ UTR (chr2: 1,299-1,824 nt) in HK1 cells. **D** Venn intersection of DEGs, DFS-associated genes, and PXDN-correlated genes of common m^6^A regulators in Fujian cohort 1. Right panel: Three candidate regulators. **E** Correlations of m^6^A regulators with PXDN expression in Fujian cohort 1. Yellow denotes candidates. **F** YTHDF1-PXDN mRNA interaction by RIP-qPCR. **G** PXDN mRNA stability assay (actinomycin D chase, 5 µg/mL) with or without YTHDF1 knockdown. **H** RNA pull-down with m^6^A-specific probe confirms YTHDF1-PXDN binding. **I** m^6^A enrichment at PXDN peak in YTHDF1-knockdown HK1 cells (MeRIP-qPCR). **J** SRAMP-predicted m^6^A motifs within PXDN 3’ UTR (NPC-specific peaks). **K** Luciferase reporter assay of wild-type versus mutant PXDN 3’ UTR in YTHDF1-knockdown HK1 cells. **L**-**O** m^6^A enrichment, PXDN expression, PXDN mRNA stability, and YTHDF1-independent regulation following m^6^A motif mutagenesis. **P**, **Q** The eliminations of YTHDF1-PXDN mRNA binding and YTHDF1-regulated mRNA stabilization effect following m^6^A motif mutation. **R** m^6^A enrichment at PXDN peak in WTAP-overexpression HK1 cells (MeRIP-qPCR). **S** PXDN mRNA stability assay (actinomycin D chase, 5 µg/mL) with or without WTAP overexpression. **T** Luciferase reporter assay of wild-type versus mutant PXDN 3’ UTR in WTAP-overexpression HK1 cells. **U** RIP-qPCR analysis of YTHDF1 occupation in PXDN with or without WTAP overexpression. *, *P* < 0.05, **, *P* < 0.01, ***, *P* < 0.001. *CDS* Coding sequences, *DEGs* Differentially expressed genes, *DFS* Disease-free survival, m^6^A N6-methyladenosine, *MeRIP* Methylated RNA immunoprecipitation, *Mut* Mutant, *NPC* Nasopharyngeal carcinoma, *qRT*-*PCR* Quantitative real-time polymerase chain reaction, *RIP* RNA immunoprecipitation, *UTR* Untranslated region, *WT* Wild-type

Epigenetic modifications critically regulate gene expression [18]. To investigate mechanisms underlying PXDN upregulation in NPC, we examined the potential involvement of m^6^A methylation-the most abundant eukaryotic mRNA modification, might be involved [19]. MeRIP sequencing revealed higher m^6^A enrichment in NPC than in normal nasopharyngeal tissues (Fig. 5A). Furthermore, multiple m^6^A sites were identified on PXDN mRNA. A region spanning 1299 to 1824 nt (3’ UTR, exon 23 of chr 2) was identified as the differential m^6^A peak (Fig. 5B). Subsequent MeRIP-qPCR validation in HK1 cells confirmed this differential methylation, suggesting m^6^A modification as a regulatory mechanism for PXDN expression (Fig. 5C).

Analysis of m^6^A regulators in the Fujian cohort 1 dataset identified three differentially expressed prognostic genes—the “reader” proteins IGF2BP3 and YTHDF1, and the “writer” WTAP—that correlated with PXDN expression (Fig. 5D). Among these, YTHDF1 exhibited the stronger association than that of IGF2BP3 (Fig. 5E). Based on this finding, we investigated a potential WTAP/YTHDF1 m^6^A axis as a mediator of PXDN dysregulation. The relationships between PXDN and other m^6^A regulators are shown in Figure S8, and these correlative expression patterns were confirmed by qRT-PCR after transient transfection. Knockdown of YTHDF1 substantially reduced PXDN expression at both transcriptional and translational levels (Figure S8A-D). RIP assay confirmed direct binding between YTHDF1 and PXDN mRNA (Fig. 5F). To assess functional redundancy among YTHDF paralogs, we observed that genetic depletion of YTHDF1 had no significant effect on YTHDF2/3 mRNA abundance but substantially reduced their protein expression (Figure S8E-F). RIP experiments indicated that neither YTHDF2 nor YTHDF3 interacted with PXDN mRNA (Figure S8G-H). Using sh-YTHDF1#1 for subsequent experiments, we observed that knockdown of YTHDF1 reduced the enrichment of PXDN mRNA (Fig. 5F) and decreased PXDN transcript stability following actinomycin D treatment (Fig. 5G), indicating YTHDF1-dependent regulation. Moreover, RNA pull-down assays revealed YTHDF1 binding to m^6^A-modified PXDN peaks (Fig. 5H). MeRIP-qPCR demonstrated diminished PXDN m^6^A methylation after YTHDF1 inhibition (Fig. 5I). Collectively, these data indicate that YTHDF1 promotes and recognizes PXDN m^6^A methylation to enhance the stability and up-regulation of PXDN in NPC.

To study the m^6^A binding site of YTHDF1 and PXDN, we employed the SRAMP algorithm, which predicted two RRACH motifs within PXDN’s differential m^6^A peak: a high-confidence (chr 2: 1,633,673) and a moderate-confidence (chr 2: 1,633,618) site (Fig. 5J). Luciferase reporter assays with a mutated high-confidence site demonstrated that YTHDF1 knockdown reduced activity exclusively in wild-type constructs (Fig. 5K), confirming the motif’s regulatory role. To test m^6^A dependence, we generated PXDN 3’ UTR mutants lacking these motifs. MeRIP-qPCR revealed significantly reduced m^6^A enrichment in mutant transcripts (Fig. 5L), which exhibited lower basal expression (Fig. 5M) and decreased stability (Fig. 5N). These findings establish that the specific m^6^A motif in PXDN is required for its m^6^A modification. The m^6^A ablation abolished YTHDF1’s regulatory effects on PXDN expression (Fig. 5O) and disrupted YTHDF1-PXDN mRNA binding (Fig. 5P). Consistent with this, the stability deficit induced by YTHDF1 knockdown was rescued in mutant transcripts (Fig. 5Q). These results conclusively demonstrate that YTHDF1 stabilizes PXDN through direct, m^6^A-dependent interactions at defined RRACH motifs.

We next probed the role of the methyltransferase WTAP in PXDN regulation. WTAP expression positively correlated with PXDN levels in NPC cells (Figure S8I-K), and its overexpression elevated both PXDN mRNA and protein abundance (Figure S8L-O). Functionally, WTAP enhanced the m^6^A methylation and transcript stability of PXDN (Fig. 5R-S). Although luciferase assays indicated that the identified RRACH motif is a primary, but not exclusive, site for WTAP-mediated regulation (Fig. 5T), WTAP overexpression robustly promoted YTHDF1 binding to PXDN mRNA (Fig. 5U). Collectively, these data establish a WTAP-YTHDF1 regulatory axis in which WTAP deposits m^6^A marks on PXDN mRNA—with a significant contribution from the identified RRACH motif—to facilitate YTHDF1 binding and subsequent transcript stabilization, thereby driving PXDN overexpression in NPC.

## Discussion

Through WGCNA analysis of EMT-related modules in NPC, we identified PXDN as a key regulator of disease progression. PXDN serves as a diagnostic marker for NPC and an independent predictor of unfavorable survival in patients. Functional assays demonstrated that PXDN drives NPC proliferation, migration, invasion, chemoradioresistance, and hepatic metastasis. Mechanistically, WTAP-mediated m^6^A methylation of PXDN mRNA, significantly facilitated by the defined RRACH motif, recruits YTHDF1 to stabilize the transcript and promote its overexpression in NPC. The upregulation of PXDN remodels the ECM to activate the oncogenic ITGB1/PI3K/AKT axis, thereby accelerating malignant progression. These results deepen our understanding of the molecular mechanisms of PXDN underlying NPC progression and suggest that PXDN represents a promising therapeutic target for clinical intervention.

PXDN, a 1,479-amino-acid secretory protein of the peroxidase XPO subfamily, is implicated in tissue development and homeostasis, with critical roles in cardiovascular disease, type 2 diabetes, and hepatocyte fibrosis [20]. Recently, PXDN has received extensive attention in oncology due to its aberrant expression correlating with tumorigenesis, progression, and therapeutic response across malignancies [10]. In breast cancer, PXDN overexpression is associated with advanced tumor grade, lymph node metastasis, and distant dissemination; patients with high PXDN expression exhibit reduced disease-free and overall survival [21]. Similarly, melanoma cell lines with invasive phenotypes show elevated PXDN levels, while PXDN silencing suppresses invasion in vitro [22, 23]. In ovarian cancer, PXDN upregulation portends poor prognosis, and its knockout attenuates proliferation, invasion, and migration [24]. Nevertheless, there are currently relatively few studies on PXDN in NPC. Only Li et al. identified PXDN as a pan-cancer diagnostic and prognostic biomarker, with validation in NPC [13]. Consistently, our current study shows that PXDN is upregulated in NPC tissues and is associated with poor prognosis. This result was also verified across multiple head and neck cancer cohorts. Functional assays demonstrated that PXDN drives aggressive phenotypes, including enhanced proliferation, migration, invasion, and metastasis. Unlike observations in rectal cancer, where PXDN downregulation occurred after preoperative radiotherapy, an irradiation dose-dependent induction of PXDN was identified in NPC [24]. Our preliminary evidence indicates that PXDN confers resistance to irradiation and cisplatin in NPC cells. It may promote therapeutic resistance through oxidative stress modulation via H₂O₂ degradation, attenuating radiotherapy-induced cytotoxicity, and ECM remodeling that enhances DNA repair through integrin-mediated signaling while simultaneously restricting chemotherapeutic penetration [25]. These findings establish PXDN as a promising therapeutic target for overcoming drug resistance in NPC. Inhibiting its peroxidase or matrix-remodeling activities may be a new strategy to improve the therapeutic effect.

Tumor cell EMT is closely related to cancer invasion and metastasis and plays a crucial role in distant dissemination [25]. Our findings establish PXDN as a multi-faceted promoter of NPC progression and metastasis, operating in part through the induction of EMT. This aligns with its previously described role in glioblastoma and underscores EMT as a conserved mechanism of PXDN-driven invasion [26]. Studies have shown that PXDN is highly expressed in cancer cells undergoing EMT, a process that underlies metastatic dissemination and poor prognosis of patients [27, 28]. Indeed, we demonstrate that PXDN overexpression in NPC induces a mesenchymal transition, characterized by upregulation of N-cadherin and vimentin with concurrent β-catenin suppression, leading to loss of epithelial polarity and enhanced invasive capacity. This oncogenic function appears conserved across malignancies, with similar EMT induction reported in cervical, melanoma, and breast cancer models [9, 23, 29, 30]. However, the regulation of β-catenin in the EMT program induced by PXDN varied across NPC cell lines. This heterogeneity likely reflects the multifaceted role of β-catenin, which functions both in cell adhesion and as a nuclear effector of Wnt signaling, with its activity being critically dependent on subcellular localization [31]. Consistent with this model, immunofluorescence analysis revealed enhanced nuclear β-catenin accumulation following PXDN overexpression in CNE2 cells, despite minimal change in its total expression. Despite this variability, PXDN was uniformly correlated with an invasive phenotype in all models examined, underscoring its non-redundant role in promoting tumour aggression.

The downstream mechanisms through which PXDN exerts its oncogenic effects have remained poorly defined. Here, we establish PXDN as an upstream activator of the PI3K/AKT pathway in NPC, a central signaling axis controlling core cancer hallmarks [32]. Previous studies in glioblastoma and ovarian cancer have noted a correlation between PXDN and PI3K/AKT signaling [12, 33]. Our functional studies establish that PI3K/AKT signaling is the principal downstream effector of PXDN-mediated oncogenicity in NPC and is necessary for PXDN-mediated EMT and cellular invasion. Rescue experiments using the specific PI3K inhibitor LY294002 demonstrate that the core functions of PXDN, including enhanced proliferation, migration and invasion, are dependent on PI3K/AKT activation, a finding consistently observed across multiple cellular models. Moreover, we utilized the proteomic analysis to demonstrate that the reduction of PXDN expression in NPC cells induced differentially expressed proteins that were mainly enriched in the ECM-receptor interaction pathway. The remodeling of ECM is closely related to the development of cancer [34]. Peroxidasin is an enzyme in the ECM. Its effects on cells are most likely through its collagen IV cross-linking activity, yet emerging evidence suggests its potential to influence cell adhesion to other ECM components [35, 36]. However, the precise mechanisms underlying these effects remain to be elucidated. In our investigation, we demonstrated that PXDN is involved in ECM remodeling, including the regulation of ECM composition and organization in NPC. Furthermore, we identify a direct physical interaction between PXDN and ITGB1, providing a mechanistic link between ECM composition and intracellular signaling. This interaction, which appears to occur through non-canonical binding motifs given the absence of RGD sequences in PXDN, promotes the assembly of integrin complexes that activate the PI3K-AKT pathway [10, 17, 37]. Further studies are warranted to explore these interactions and mechanisms in greater detail.

While PXDN dysregulation has been implicated in tumor progression, the molecular mechanisms governing its expression remain incompletely understood. PXDN expression is regulated by a diverse array of activators, including proinflammatory stimuli and metabolic factors. Intriguingly, folic acid has been shown to suppress PXDN expression through promoter DNA methylation [38]. Oxidative stress mediators (H_2_O_2_, tert-butylhydroquinone, sulforaphane) activate PXDN via the Nrf2 pathway in HeLa and HEK293 cells [39]. Additionally, post-transcriptionally, PXDN mRNA undergoes 2’-O-methylation guided by snoRNAs U32A and U51 [40]. Our investigation establishes an epitranscriptional basis for PXDN dysregulation in NPC. We first identified elevated m^6^A levels in NPC tissues and specific m^6^A site in PXDN mRNA. We subsequently delineated a WTAP/YTHDF1 axis as the central regulator: WTAP deposits the m^6^A mark on PXDN transcripts, which are then recognized by YTHDF1, enhancing both mRNA stability and translational elongation. WTAP may be superior to METTL3/METTL14 in regulating PXDN, implying distinct substrate recognition or context-dependent roles for writer complexes in NPC. Similarly, the specificity of this axis is underscored by our finding that YTHDF1 is the principal m^6^A reader for PXDN; its paralogs, YTHDF2 and YTHDF3, neither bind the transcript nor compensate for its loss. While this dedicated axis constitutes the core epitranscriptional mechanism, the broader m^6^A network may provide auxiliary regulatory layers in different pathological contexts. Future studies exploring the potential contribution of additional m^6^A sites or modifiers, and their crosstalk with known regulators such as Nrf2, will further unravel the sophisticated control of oncogenic PXDN expression. Furthermore, the mechanistic model proposed here is derived primarily from studies in HK1 cells. Although this offers a well-defined system for initial discovery, the generalizability of this epitranscriptional regulatory mechanism across the diverse molecular subtypes of NPC remains to be fully elucidated and represents an essential direction for subsequent investigation.

**Fig. 6:**
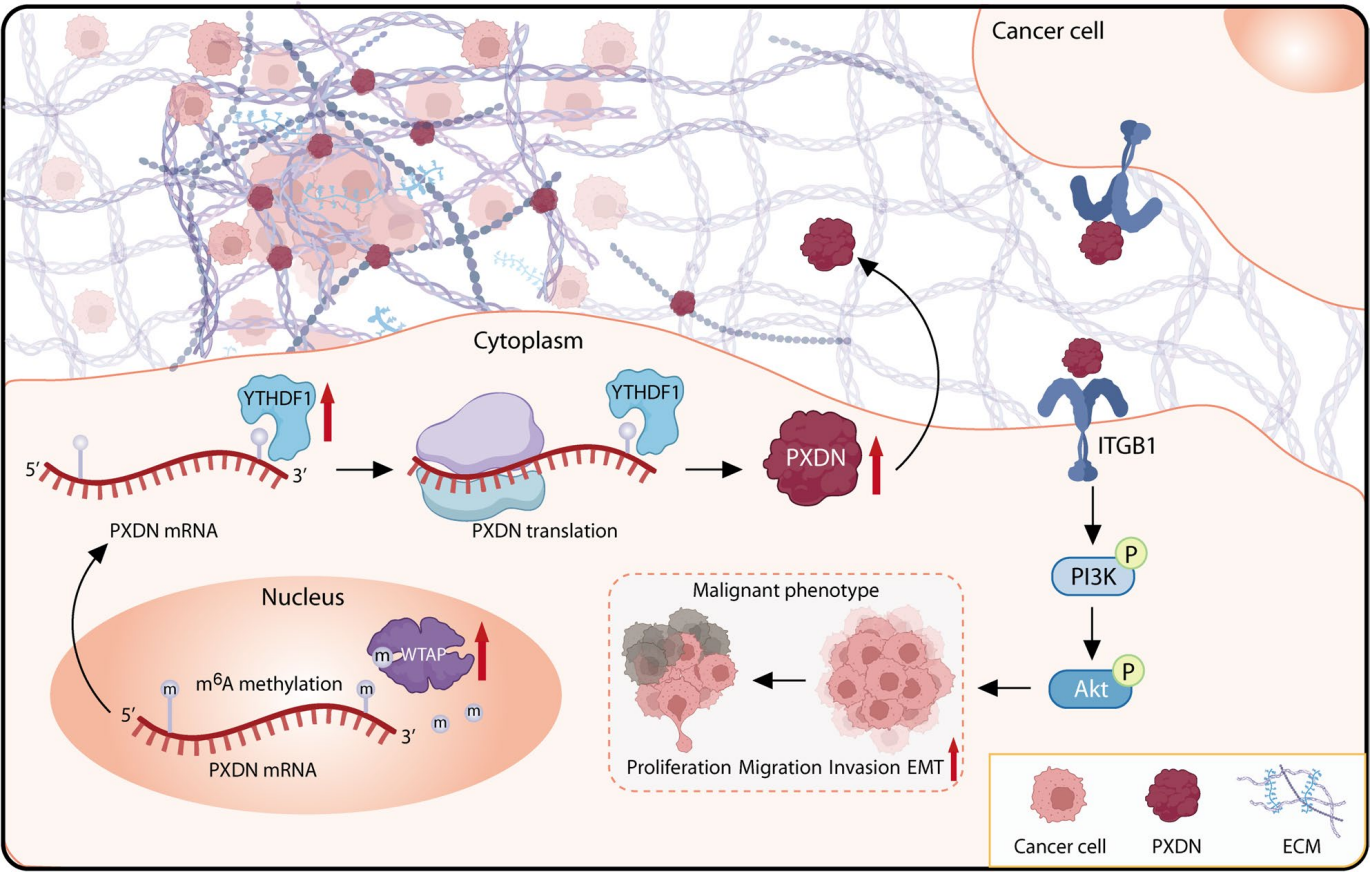
Proposed mechanism by which PXDN promotes the malignant phenotypes in NPC. YTHDF1 recognizes WTAP-mediated m^6^A-modified PXDN mRNA, enhancing its stability and translation. Up-regulated PXDN expression drives malignant progression of NPC by activating ECM remodeling to induce the oncogenic ITGB1/PI3K/AKT pathway. *ECM* Extracellular matrix; *EMT* Epithelial-mesenchymal transition, m^6^A N6-methyladenosine, *NPC* Nasopharyngeal carcinoma

In conclusion, this study demonstrates the important role of PXDN in NPC progression. YTHDF1 recognizes WTAP-mediated m^6^A methylation to facilitate stability and translation of PXDN, leading to its aberrant up-regulation in NPC. Up-regulated PXDN expression further promotes malignant phenotype of NPC by activating ECM remodeling to induce the oncogenic ITGB1/PI3K/AKT pathway (Fig. 6). These findings provide insights into the molecular basis of NPC progression and suggest avenues for the development of predictive biomarkers and targeted therapeutic strategies.

## Supplementary Information

Supplementary Material 1.

## Data Availability

Data of the research is available upon reasonable request from the corresponding author.

## Abbreviations

Co-IP: Co-immunoprecipitation
DEGs: Differentially expressed genes
DFS: Disease-free survival
ECM: Extracellular matrix
EMT: Epithelial-mesenchymal transition
IC_50_: Half maximal inhibitory concentration
IHC: Immunohistochemical
KEGG: Kyoto Encyclopedia of Genes and Genomes
m^6^A: N6-methyladenosine
MeRIP: Methylated RNA immunoprecipitation
NPC: Nasopharyngeal carcinoma
PXDN: Peroxidasin
qRT-PCR: Quantitative real-time polymerase chain reaction
RIP: RNA immunoprecipitation
RSI: Radiation sensitivity index
WGCNA: Weighted gene co-expression network analysis
