# Supplementary material for: PXDN regulated by WTAP/YTHDF1-mediated m6A modification activates PI3K/AKT signaling pathway through extracellular matrix remodeling to promote progression in nasopharyngeal carcinoma
Source: J Exp Clin Cancer Res. 2025 Dec 18;45:21. doi: 10.1186/s13046-025-03609-y (PMC12825198; doi:10.1186/s13046-025-03609-y)
Supplement: Supplementary file 1 — Supplementary Material 1. [file 13046_2025_3609_MOESM1_ESM.pdf]

Supplementary Methods

Public datasets

Gene expression profiles of nasopharyngeal carcinoma (NPC) and head and neck cancer (HNSC) were acquired from the Gene Expression Omnibus (GEO) (http://www.ncbi.nlm.nih.gov/geo), The Cancer Genome Atlas (TCGA) (https://portal.gdc.cancer.gov/) and ArrayExpress (https://www.ebi.ac.uk/arrayexpress/) for external validation. The analysis incorporated multiple independent cohorts: GSE12452 (31 NPC, 10 normal) [1], GSE53819 (18 NPC, 18 normal) [2], and GSE118719 (7 NPC, 4 normal) [3] for NPC; alongside TCGA-HNSC (522 HNSC, 42 normal) [4], GSE42743 (29 HNSC, 74 normal) [5], GSE75538 (14 tumor-normal pairs) [6], and E-MTAB-8588 (117 HNSC, 98 normal) for HNSC. For prognostic analysis, two independent cohorts were utilized: the GSE102349 dataset, comprising 113 NPC patients, and the GSE117973 cohort, consisting of 77 HNSC patients [7]. Differential expression analysis was performed using the "limma" R package, with |fold change (FC)| > 0.5 and adjusted P value < 0.05 as thresholds for identifying differentially expressed genes (DEGs).

Single-sample gene set enrichment analysis

Epithelial-mesenchymal transition (EMT) scores were assessed by single-sample gene set enrichment analysis (ssGSEA), based on the HALLMARK_EPITHELIAL_MESENCHYMAL_TRANSITION gene set from the Molecular Signatures Database (MSigDB) and implemented using the "GSVA" and "GSEABase" R packages. The radiosensitivity index (RSI), an inverse indicator of tumor radiosensitivity, was derived as previously described [8]. Drug sensitivity for each patient was predicted from bulk RNA-seg data using the "oncoPredict" R package [9].

RNA isolation, library construction, and sequencing

Total RNA was extracted using TRIzol reagent (Invitrogen, USA) following the manufacturer's protocol. Poly(A)-enriched mRNA was isolated from NPC cells using oligo(dT)-coupled magnetic beads. The purified mRNA was fragmented and reverse-transcribed into cDNA. The resulting double-stranded cDNA was end-repaired, adenylated at the 3' ends, and ligated with sequencing adapters. Polymerase chain reaction (PCR) amplification was performed to generate the final cDNA library, which was sequenced by OE Biotech (China).

Western blotting analysis

Proteins were extracted using RIPA lysis buffer (Abcam, USA) and separated on 4–12% SmartPAGE™ Precast Gels (Tiandirenhe Biotechnology, China). After electrophoresis, proteins were transferred to a 0.45 μm PVDF membrane (Beyotime Biotechnology, China). After blocking with QuickBlock™ buffer (Beyotime Biotechnology, China) for 10 min at room temperature, the membrane was probed with primary antibodies (see **Table S1**) overnight at 4 °C. HRP-conjugated secondary

antibodies were applied for 1 h at room temperature, and protein bands were visualized using an ECL detection kit (MedChemExpress, USA).

RNA extraction and quantitative reverse transcription PCR (qRT-PCR)

Total RNA was isolated using the RNAeasy™ kit (Beyotime Biotechnology, China) and reverse-transcribed with the Eastep® RT Master Mix (Promega, China). qRT-PCR was performed with Eastep® qPCR Master Mix (Promega, China) on an Applied Biosystems 7500 FAST system (Thermo Fisher Scientific, USA). Gene-specific primers (**Table S2**) were used, with GAPDH as the endogenous control. Relative mRNA expression was calculated via the $2^{-\Delta\Delta Ct}$ method, normalized to the control group, and presented as fold change.

Transient transfection and stable cell line generation

To identify optimal PXDN knockdown sites, cells at 60-80% confluency were transiently transfected with PXDN-targeting or non-targeting scrambled siRNA (negative control; GenePharma, China) using Lipofectamine 3000 (invitrogen, USA). The medium was replaced after 6-8 h, and cells were harvested for verification 48-72 h post-transfection. For stable genetic manipulation, cells at 30-40% confluency were infected with lentiviral vectors at multiplicities of infection (MOI) of 5 (HK1) or 10 (CNE2, C666-1) in medium supplemented with 1 mg/mL polybrane. The MOI was calculated as: (cell number × 1000) / lentiviral titer (TU/mL). Infection efficiency was monitored by fluorescence microscopy (Olympus, Japan) 48-72 h later, and stable polyclonal populations were selected with puromycin (1 μg/mL for HK1; 2 μg/mL for CNE2 and C666-1) for 10-14 days. Knockdown and overexpression efficiency were validated by qRT-PCR and immunoblotting.

Cell proliferation assays

For the cell viability assay, cells (HK1: 3,000; CNE2: 2,000; C666-1: 3,000 per well) were seeded in 96-well plates and incubated at 37 °C under 5% CO_2. Following adhesion, viability was assessed every 24 h by adding 10% cell counting kit-8 (CCK-8) solution (MedChemExpress, USA) per well, incubating for 2 h at 37 °C, and measuring absorbance at 450 nm using a multi-scan spectophotometer (Infinite® 200 Pro, Tecan).

For colony formation assay, cells (3,000 per well) were plated in 6-well plates and cultured (HK1: 7-14 days; CNE2: 4-7 days; C666-1: 14-21 days) until visible colonies formed. Colonies were fixed with 4% paraformaldehyde (Beyotime Biotechnology, China) for 15 min, stained with crystal violet (Beyotime Biotechnology, China) for 15 min, photographed, and quantified using ImageJ software.

To assess DNA synthesis rates, cells (1×10^5 per well) were seeded in 24-well plates. DNA synthesis was measured using the Click-iT EDU Imaging Kit (Beyotime Biotechnology, China) according to the manufacturer's protocol.

Migration and invasion assays

Cell migration and invasion were assessed using Transwell inserts (8 μm pore size;

Corning, USA) pre-coated with or without Matrigel (50μL of 1:8 diluted, Corning, USA). Briefly, 1 × 10^5 cells in 100 μL serum-free medium were seeded into the upper chamber, while the lower chamber contained 500 μL medium supplemented with 20% fetal bovine serum (FBS, Gibco, USA). Following incubation at 37 ℃ for the indicated times (HK1: 72 h; CNE2: 18 h; C666-1: 96 h), non-migrated cells were washed with phosphate-buffered saline (PBS, BasalMedia, China), and migrated cells were fixed with 4% paraformaldehyde, stained with crystal violet, and imaged.

Wound healing assay

Cell migration was further evaluated using a scratch-wound assay. Cells were seeded in 24-well plates and grown to confluence. A linear scratch was made with a P-200 pipette tip, and the wells were washed with PBS to remove debris. Cells were then maintained in serum-free medium. Wound closure was monitored every 6 h, and migration rates were quantified by measuring the reduction in wound area over time.

Immunohistochemistry

Formalin-fixed, paraffin-embedded tissues were sectioned at 4 μm thickness and mounted on slides. Sections were incubated overnight at 4 °C with primary antibodies against PXDN (1 μg/mL; Invitrogen, USA) and Ki67 (1:5,000; Proteintech, USA), following established protocols [10]. Two blinded independent pathologists evaluated staining intensity using a standardized histoscore system.

Immunofluorescence

Cells adhered to confocal dishes were fixed in 4% paraformaldehyde, permeabilized with 0.5% Triton X-100 (Beyotime Biotechnology, China), and blocked prior to overnight incubation at 4 °C with target-specific primary antibodies. Fluorophore-conjugated secondary antibodies (Alexa Fluor 488; Beyotime Biotechnology, China) were applied for 1 h in darkness, followed by nuclear counterstaining with DAPI. Images were acquired on a confocal microscope (Zeiss LSM 980, Germany).

Irradiation and cisplatin treatment

Cells were cultured in 6-well plates for X-ray treatment using an Elekta Synergy linac, with harvesting 48 h post-exposure. Irradiation doses of 2, 4, 6, and 8 Gy were administered at a dose rate of 2 Gy/min. For cisplatin treatment (Nuoxin injection, Hansoh Pharmaceutical Group, China), cells (HK1: seeded at 3 × 10^4; CNE2: 2 × 10^4 cells; C666-1: 4 × 10^4 cells per well, respectively) were incubated in 96-well plates with cisplatin concentrations ranging from 0 to 25 μg/mL for 48 h. Cell viability was assessed using CCK-8 solution, and proliferation, apoptosis, and cell cycle assays were performed following reagent or irradiation exposure.

DNA double-strand break (DSB) analysis

DSBs were analyzed via γ-H2AX immunostaining with a DNA Damage Assay Kit (Beyotime Biotechnology, China), adhering to the manufacturer's protocol. The

operation is the same as that of the immunofluorescence.

Flow cytometry analysis of apoptosis and cell cycle

Apoptosis was assessed using a PE Annexin V Apoptosis Detection Kit (BD Pharmingen, USA). Cells were harvested 48 h post-treatment with cisplatin or irradiation, washed with PBS, and resuspended in 500 μL binding buffer before staining with 10 μL Annexin V-APC and 5 μL 7-AAD. For cell cycle analysis, ethanol-fixed cells were washed and stained with propidium iodide (Cell Cycle Staining Kit; Beyotime Biotechnology, China) for 30 min at room temperature in the dark. Samples were analyzed on a Beckman CytoFLEX flow cytometer (USA). Apoptosis rates were quantified using CytExpert software (v2.4; Beckman Coulter, USA), and cell-cycle distributions were modeled with ModFit (v4.1.7; Verity Software House, USA).

Mass spectrometry analysis

Protein identification was performed by LC-MS/MS using a Q Exactive Plus mass spectrometer coupled with an Easy nLC system (Thermo Fisher Scientific, USA). In brief, samples were lysed in buffer containing protease inhibitors, digested via the FASP method, and peptides were solubilized in 0.1% formic acid prior to analysis. Data were processed using Proteome Discoverer 2.2 and Mascot 2.6.

Proteomic profiling

Total proteins were extracted, quantified, and verified by SDS-PAGE. Trypsin-digested peptides were desalted and analyzed by LC-MS/MS (timsTOF HT, Bruker) using data-independent acquisition. Raw spectra were matched against reference databases, retaining proteins with ≥1 unique peptide and ≥ 2 valid values across replicates (≥ 50% valid values per group). Missing values were imputed using group means (for proteins with ≥ 50% valid values) or half-minimum values. Data were median-normalized and log2-transformed. Sample reproducibility and group separation were assessed by principal component analysis and hierarchical clustering. Differential expressed proteins were identified using the "edgeR" package (threshold: $|log2FC| > 1$, $P < 0.05$) and visualized via volcano plots.

MeRIP sequencing (MeRIP-Seq)

MeRIP sequencing was performed (LC Bio, China) following established protocols [11]. Total RNA was isolated using TRIzol reagent, with integrity verified on a Bioanalyzer 2100 (Agilent, USA) and concentration quantified via NanoDrop ND-1000 (NanoDrop, USA). Poly(A) RNA was specifically enriched using oligo(dT) magnetic beads (Thermo Fisher Scientific, USA). Samples with > 50 μg total RNA underwent fragmentation and subjected to immunoprecipitation with Dynabeads (Thermo Fisher Scientific, USA) and an anti-m^6A antibody (Synaptic Systems, 202003). Immunoprecipitated RNA was reverse-transcribed, and cDNA libraries were prepared through end-repair, A-tailing, UDG treatment (NEB, m0280), and PCR amplification. Parallel input libraries were constructed with the TruSeq Stranded

mRNA Kit (Illumina, USA). All libraries were sequenced (PE 150) on an Illumina Novaseq 6000 (Hangzhou, China). Adaptor-trimmed and quality-filtered reads (fastp, v0.19.4) [12] were aligned to GRCh38/hg38 (HISAT2 v2.2.1) [13]. Differentially methylated peaks were identified by "exomePeak" package [14] and annotated by ANNOVAR [15].

Inhibitor treatment regimens

The half-maximal inhibitory concentrations (IC_{50}) of phloroglucinol (Sigma-Aldrich, Merk, Germany) [16] were determined via cell proliferation assays. The following optimized inhibitor concentrations were used in this study: Phloroglucinol: 9 mM for HK1 and CNE2 cells and 8 mM for C666-1 cells; LY294002 (MedChemExpress, USA) [17,18]: 20 μM; and Cilengitide (MedChemExpress, USA) [19]: 15 nM.

Reference

1. Sengupta S, den Boon JA, Chen IH, et al. Genome-wide expression profiling reveals EBV-associated inhibition of MHC class I expression in nasopharyngeal carcinoma. Cancer Res. 2006;66(16):7999-8006.
2. Bao YN, Cao X, Luo DH, et al. Urokinase-type plasminogen activator receptor signaling is critical in nasopharyngeal carcinoma cell growth and metastasis. Cell Cycle. 2014;13(12):1958-1969.
3. Lin C, Zong J, Lin W, et al. EBV-miR-BART8-3p induces epithelial-mesenchymal transition and promotes metastasis of nasopharyngeal carcinoma cells through activating NF-κB and Erk1/2 pathways. J Exp Clin Cancer Res. 2018;37(1):283.
4. Thorsson V, Gibbs DL, Brown SD, et al. The immune landscape of cancer. Immunity. 2018;48(4):812-30.e14.
5. Lohavanichbutr P, Méndez E, Holsinger FC, et al. A 13-gene signature prognostic of HPV-negative OSCC: discovery and external validation. Clin Cancer Res. 2013;19(5):1197-1203.
6. Krishnan NM, Dhas K, Nair J, et al. A minimal DNA methylation signature in oral tongue squamous cell carcinoma links altered methylation with tumor attributes. Mol Cancer Res. 2016;14(9):805-819.
7. Zhang L, MacIsaac KD, Zhou T, et al. Genomic analysis of nasopharyngeal carcinoma reveals TME-based subtypes. Mol Cancer Res. 2017;15(12):1722-1732.
8. Robb R, Yang L, Shen C, et al. Inhibiting BRAF oncogene-mediated radioresistance effectively radiosensitizes BRAFV600E-mutant thyroid cancer cells by constraining DNA double-strand break repair. Clin Cancer Res. 2019;25(15):4749-4760.
9. Maeser D, Gruener RF, Huang RS. oncoPredict: an R package for predicting in vivo or cancer patient drug response and biomarkers from cell line screening data. Brief Bioinform. 2021;22(6):bbab260.
10. Huang Z, Li Y, Liu Q, et al. SPP1-mediated M2 macrophage polarization shapes

the tumor microenvironment and enhances prognosis and immunotherapy guidance in nasopharyngeal carcinoma. Int Immunopharmacol. 2025;147:113944.

11. Zhang P, Zhang W, Wang X, et al. BCLAF1 drives esophageal squamous cell carcinoma progression through regulation of YTHDF2-dependent SIX1 mRNA degradation. Cancer Lett. 2024;591:216874.
12. Chen S, Zhou Y, Chen Y, Gu J. fastp: an ultra-fast all-in-one FASTQ preprocessor. Bioinformatics. 2018;34(17):i884-i890.
13. Kim D, Paggi JM, Park C, et al. Graph-based genome alignment and genotyping with HISAT2 and HISAT-genotype. Nat Biotechnol. 2019;37(8):907-915.
14. Meng J, Lu Z, Liu H, et al. A protocol for RNAmethylation differential analysis with MeRIP-Seq data and exomePeak R/Bioconductor package. Methods. 2014;69:274-281.
15. Wang K, Li M, Hakonarson H. ANNOVAR: Functional annotation of genetic variants fromnext-generation sequencing data. Nucleic Acids Research. 2010;38(16):e164.
16. Paumann-Page M, Obinger C, Winterbourn CC, Furtmüller PG. Peroxidasin inhibition by phloroglucinol and other peroxidase inhibitors. Antioxidants (Basel). 2023;13(1):23.
17. Huang A, Zeng P, Li Y, et al. LY294002 is a promising inhibitor to overcome sorafenib resistance in FLT3-ITD mutant AML cells by interfering with PI3K/Akt signaling pathway. Front Oncol. 2021;11:782065.
18. Huang Z. Exploring the role and mechanism of SPP1 in nasopharyngeal carcinoma progression via the PI3K/AKT signaling pathway [D]. Fujian Medical University. 2024.
19. Kapp TG, Rechenmacher F, Neubauer S, et al. A comprehensive evaluation of the activity and selectivity profile of ligands for RGD-binding integrins. Sci Rep. 2017;7:39805.

Table S1. List of antibodies used in this study

Antibodies	Source	Cat#	Dilution ratio
PXDN	Invitrogen	PA5-144080	0.5 μg/mL for WB; 1 μg/mL for IHC
PXDN	Santa Cruz	sc-293408	5 μg for IP
IgG	MedChemExpress	HY-P80879	5 μg for IP; 10 μg for MeRIP
Anti-m^6A	Abcam	ab208577	10 μg for MeRIP
N-cadherin	Proteintech	66219-1-Ig	1:10000 dilution for WB; 1:400 dilution for IF
β catenin	Proteintech	66379-1-Ig	1:10000 dilution for WB; 1:400 dilution for IF
Vimentin	Proteintech	60330-1-Ig	1:50000 dilution for WB; 1:1000 dilution for IF
Snail	Cell Signaling Technology	3879	1:1000 dilution for WB
PI3K	Cell Signaling Technology	4292	1:3000 dilution for WB
Phospho-PI3K	Cell Signaling Technology	17366	1:3000 dilution for WB
Akt	Cell Signaling Technology	4691	1:3000 dilution for WB
Phospho-Akt	Cell Signaling Technology	4060	1:3000 dilution for WB
ITGB1	Proteintech	12594-1-AP	1:10000 dilution for WB
ATP1A1	Proteintech	68735-2-Ig	1:10000 dilution for WB
GST Tag	Proteintech	66001-2-Ig	1:10000 dilution for WB
WTAP	Proteintech	60188-1-Ig	1:10000 dilution for WB; 10 μg for MeRIP
YTHDF1	Proteintech	66745-1-Ig	1:3000 dilution for WB; 5 μg for RIP
YTHDF2	Selleck	F2153	1:1000 dilution for WB; 5 μg for RIP
YTHDF3	Selleck	F2793	1:1000 dilution for WB; 5 μg for RIP
GAPDH	Proteintech	HRP-60004	1:50000 dilution for WB
Anti-Rabbit IgG	Cell Signaling Technology	7074	1:3000 dilution for WB
Anti-Mouse IgG	Cell Signaling Technology	7076	1:3000 dilution for WB
Ki67	Proteintech	66555-6-Ig	1:5000 dilution for IHC
Anti-Mouse AF488	Beyotime	P0188-1	1:1000 dilution for IF

IF, immunofluorescence; IHC, immunohistochemistry; IP, immunoprecipitation; m^6A, N6-methyladenosine; MeRIP, Methylated RNA immunoprecipitation; RIP, RNA immunoprecipitation; WB, western blotting.

Table S2. qRT-PCR primers used in this study

Gene	Forward (5' to 3')	Reverse (5' to 3')
PXDN	AGGACTGCTGTGAAGACTGTA	GGTGCTGTTGCTGAGATGTT
PXDN-MeRIP	CCTCTGGGCTCTCTGTAACA	AATCAGGTGTGGAAGGAGCT
PC-h-PXDN	GAGCAGTAATTCTAGGCGATCGCTC	AAGATATTTTATTGCGGCCAGCGGCC
3' UTR-wt	GAGGCTCCTGGGAGGCTCCTCAG	GCTCCATTTGAAAATGTGACCCA
PC-h-PXDN Cas9-gRNA1	CACCgGCGTTCACAGGGCCACTGTC	AAACGACAGTGGCCCTGTGAACGCc
PC-h-PXDN Cas9-gRNA2	CACCgTGCACTGCACCCCCAGACAG	AAACCTGTCTGGGGGTGCAGTGCAc
PC-h-PXDN Cas9-gRNA3	CACCgGGGGGTGCAGTGCAAGCCCC	AAACGGGGCTTGCACTGCACCCCCc
YTHDF1	CGACATCCACCGCTCCATTA	GCTGAAGAGCAGGTAGACGG
WTAP	AATCCAGTACCTCAAGCAAGTC	TGTCTTTAGTCTGTTCCAGTTCAC
RBM15B	TAACCTGGACCACAGCGTAT	TCTGGAACTTGAGGAAGGCATA
ZC3H13	AGATGACGAGTCCAAGTTAGATGA	GGCATAAGACCAGACCAATCC
ALKBH5	GCGCAAGTATCAGGAGGACT	CGTTGTACAGGCCCTTCTCA
YTHDC1	AACTGGTTTCTAAGCCACTGAGC	GGAGGCACTACTTGATAGACGA
YTHDF2	TTGCAGGGTCAAGTCTTCCG	CCTACTGATGCCTGAGCCAC
YTHDF3	TGTTGTGGACTATAATGCGTATGC	AAGCGAATATGCCGTAATTGGTTA
IGF2BP2	GCAAGACCGTGAACGAACTG	CCGATAATTCTGACGATCACTTCC
IGF2BP3	GCTCATATCAGAGTGCCATCC	TGGTCATTCTCATCAGGTGTCT
HNRNPA2B1	TGGTGGTAGCAGGAACATGG	TCAGTATCGGCTCCTCCCAC
Aggrecan	GTGCCTATCAGGACAAGGTCT	GATGCCTTTCACCACGACTTC
COL1A1	GAGAGCATGACCGATGGATTC	CTTCTTGAGGTTGCCAGTCTG
COL4A1	GGGTCGGAGAGAAAGGTGAA	GGTCCTGTGCCTATAACAATTCC
FN1	AGACCAGCAGAGGCATAAGG	CCACTCATCTCCAACGGCATA
MMP3	CCATCTCTTCCTTCAGGCGT	CTCACGGTTGGAGGGAAACC
MMP13	CGCCAGACAAATGTGACCCT	GCAGCATCAATACGGTTGGG
ITGB1	GACAAATTACCCCAGCCGGT	AGCAACCACACCAGCTACAA
GAPDH	GGTGTGAACCATGAGAAGTATGA	GAGTCCTTCCACGATACCAAAG

gRNA, guide RNA; MeRIP, methylated RNA immunoprecipitation; qRT-PCR, quantitative real-time polymerase chain reaction; UTR, untranslated region; wt, wild-type.

Table S3. The oligonucleotide sequences used in this study.

Gene	Forward (5' to 3')	Reverse (5' to 3')
si-NC	UUCUCCGAACGUGUCACGUTT	ACGUGACACGUUCGGAGAATT
si-PXDN (559)	CAACCGGAUUACACAUUUATT	UAAAUGUGUAAUCCGGUUGTT
si-PXDN (892)	CUGGCUGCGAAACAAUAAUTT	AUUAUUGUUUCGCAGCCAGTT
**si-PXDN (1242)**	**GCGGGCUUUACAUACAGAATT**	**UUCUGUAUGUAAAGCCCGCTT**
**si-WTAP (368)**	**CUAAGAGAGUCUGAAGAATT**	**UUCUUCAGACUCUCUUAGGTT**
si-WTAP (421)	GGGAAAACAUCCUUGUAAUTT	AUUACAAGGAUGUUUUCCCTT
si-WTAP (590)	GGAACAGACUAAAGACAAATT	UUUGUCUUUAGUCUGUUCCTT
si-RBM15B (1203)	UCGAGGAGGUGGUCAUCAATT	UUGAUGACCACCUCCUCGATT
si-RBM15B (2147)	GGGAAGAAGGCAAGAGACATT	UGUCUCUUGCCUUCUUCCCTT
**si-RBM15B (2734)**	**GGGCAAGCUAGAAGAAGAATT**	**UUCUUCUUCUAGCUUGCCCTT**
si-ZC3H13 (692)	CUAAGAAAGUCUAGCAAAUTT	AUUUGCUAGACUUUCUUAGTT
**si-ZC3H13 (2378)**	**CGAGAAAGAGCGAGAGAAATT**	**UUUCUCUCGCUCUUUCUCGTT**
si-ZC3H13 (3992)	GAGAGAGAGAGGAGAGAUATT	UAUCUCUCCUCUCUCUCUCTT
si-ALKBH5 (580)	GGGCCAAGCGCAAGUAUCATT	UGAUACUUGCGCUUGGCCCTT
**si-ALKBH5 (1102)**	**GCUGCAAGUUCCAGUUCAATT**	**UUGAACUGGAACUUGCAGCTT**
si-ALKBH5 (1187)	GAUAUGCUGCUGAUGAAAUTT	AUUUCAUCAGCAGCAUAUCTT
si-YTHDC1 (358)	GGAGAAAGAUGGAGAACUUTT	AAGUUCUCCAUCUUUCUCCTT
si-YTHDC1 (1240)	GAAGAGAGCUAGAGGCAUATT	UAUGCCUCUAGCUCUCUUCTT
**si-YTHDC1 (2216)**	**GAAGCAAGAUACAGAGAUATT**	**UAUCUCUGUAUCUUGCUUCTT**
si-YTHDF2 (699)	CAUGAAUACUAUAGACCAATT	UUGGUCUAUAGUAUUCAUGTT
si-YTHDF2 (1712)	AGAAUAAACCAGUGACCAATT	UUGGUCACUGGUUUAUUCUTT
**si-YTHDF2 (1875)**	**ACGUCAAGGUCGUGGGAAATT**	**UUUCCCACGACCUUGACGUTT**
si-YTHDF3 (356)	ACAAAUGAGUAAUGGAGAATT	UUCUCCAUUACUCAUUUGUTT
**si-YTHDF3 (1577)**	**GGGCAAAUUUGAAGUUAAATT**	**UUUAACUUCAAAUUUGCCCTT**
si-YTHDF3 (1817)	GGAGAGAAAUAGAAACAAATT	UUUGUUUCUAUUUCUCUCCTT
**si-IGF2BP2 (699)**	**CAAACUAGCCGAAGAGAUUTT**	**AAUCUCUUCGGCUAGUUUGTT**
si-IGF2BP2 (771)	AGGCAGAAAUUUGAAGAAATT	UUUCUUCAAAUUUCUGCCUTT
si-IGF2BP2 (899)	GUGCUGAGAUAGAGAUUAUTT	AUAAUCUCUAUCUCAGCACTT
si-IGF2BP3 (1099)	GCUGAGAAGUCGAUUACUATT	UAGUAAUCGACUUCUCAGCTT
si-IGF2BP3 (1400)	AGGAGGAGAUCAUGAAGAATT	UUCUUCAUGAUCUCCUCCUTT
**si-IGF2BP3 (1782)**	**GGGAAGAAUUUAUGGAAAATT**	**UUUUCCAUAAAUUCUUCCCTT**
si-HNRNPA2B1 (231)	CAGAAGAAAGUUUGAGGAATT	UUCCUCAAACUUUCUUCUGTT
**si-HNRNPA2B1 (396)**	**GGAGAGUAGUUGAGCCAAATT**	**UUUGGCUCAACUACUCUCCTT**
si-HNRNPA2B1 (519)	GAGAUUACUUUGAGGAAUATT	UAUUCCUCAAAGUAAUCUCTT

Bold font indicates the optimal knockdown sequence.

Table S4. Easy-siRNA design for YTHDF1.

Gene	NO.	Target Seq
sh-NC	CON313	TTCTCCGAACGTGTCACGT
**sh-YTHDF1#1**	**PSC119750**	**GGATACAGTTCATGACAATGA**
sh-YTHDF1#2	PSC119751	GCACTGACTGGTGTCCTTTCT
sh-YTHDF1#3	PSC119752	GGCGTGTGTTCATCATCAAGA

Bold font indicates the optimal knockdown sequence.

Table S5. Probes used for RNA pull-down in this study.

**Probe Name**	**Probe Type**	**Sequence**
Pos	Positive Probe	5’ Biotin-TGGTTGCGTCCACGTCCTGAACAAGAGCCGCTT 3’
Neg	Negative Probe	5’ Biotin-AAGCGGCTCTTGTTCAGGACGTGGACGCAACCA 3’

Table S6. Sequences of cDNA fused in Luciferase reporters

Type	Sequence
Wild-type cDNA	AGGTGTGAGGTGGGAGGATGTGTCCAGCTGGTGGTCTTGGCCCCTCAGGGTG<br>AGAGGACCGTGGGCATGGACGGAAGGGTGGGACGGGAGCCTCAGGGAGACT<br>TGTTATGGGGGCTCCCATTAACTCATACCTGTCTGCAACTATGAAATCCTAAG<br>AGAACCACAAAATCCATGCCCACAGGGCAAGTACAGCGGGCAAGGCAGGGG<br>CCTCAGGGCAAGGCCTGACTCATGGCCGTGGCTGACACCTGTCCCGGAGCTG<br>ACAGGCTTTGCTCACCTGAAGGCCATCCCAGAGCTGACAGGCTGTGCTCCGT<br>CTCTCTTCTCCAGGACGGGCAGGTCACCTGCTTCGTGGAAGCTTGCCCCCCTG<br>CCACCTGTGCTGTCCCCGTGAACATCCCAGGGGCCTGCTGTCCAGTCTGCTTA<br>CAGAAGAGGGCGGAGGAAAAGCCCTAGGCTCCTGGGAGGCTCCTCAGAGTTT<br>GTCTGCTGTGCCATCGTGAGATCGGGTGGCCGATGGCAGGGAGCTGCGGACT<br>GCAGACCAGGAAACACCCAGAACTCGTGACATTTCATGACAACGTCCAGCTG<br>GTGCTGTTACAGAAGGCAGTGCAGGAGGCTTCCAACCAGAGCATCTGCGGAG<br>AAGGAGGCACAGCAGGTGCCTGAAGGGAAGCAGGCAGGAGTCCTAGCTTCA<br>CGTTAGACTTCTCAGGTTTTATTTAATTCTTTTAAAATGAAAAATTGGTGCTA<br>CTATTAAATTGCACAGTTGAATCATTTAGGCGCCTAAATTGATTTTGCCTCCC<br>AACACCATTTCTTTTTAAATAAAGCAGGATACCTCTATATGTCAGCCTTGCCT<br>TGTTCAGATGCCAGGAGCCGGCAGACCTGTCACCCGCAGGTGGGGTGAGTCT<br>TGGAGCTGCCAGAGGGGCTCACCGAAATCGGGGTTCCATCACAAGCTATGTT<br>TAAAAAGAAAATTGGTGTTTGGCAAACGGAACAGAACCTTTGATGAGAGCGT<br>TCACAGGGACACTGTCTGGGGGTGCAGTGCAAGCCCCCGGCCTCTTCCCTGG<br>GAACCTCTGAACTCCTCCTTCCTCTGGGCTCTCTGTAACATTTCACCACACGT<br>CAGCATCTAATCCCAAGACAAACATTCCCGCTGCTCGAAGCAGCTGTATAGC<br>CTGTGACTCTCCGTGTGTCAGCTCCTTCCACACCTGATTAGAACATTCATAAG<br>CCACATTTAGAAACAGGTTTGCTTTCAGCTGTCACTTGCACACATACTGCCTA<br>GTTGTGAACCAAATGTGAAAAAACCTCCTTCATCCCATTGTGTATCTGATACC<br>TGCCGAGGGCCAAGGGTGTGTGTTGACAACGCCGCTCCCAGCCGGCCCTGGT<br>TGCGTCCACGTCCTGAACAAGAGCCGCTTCCGGATGGCTCTTCCCAAGGGAG<br>GAGGAGCTCAAGTGTCGGGAACTGTCTAACTTCAGGTTGTGTGAGTGCGTTA<br>AAAAAAAAAAAAAAAAAGAATCCCTATACCTCATTTGTATTTTTAAAATGCG<br>TGATGTTTTATGAAATTGTGTCCATTTTTTAGGTATTAGATATGGCAGAAAAA<br>CCATTTCCACTATGCAAAGTTCTTTTAGACGTCAGTGAAAATCAACTCTCATA<br>CCTCATGGTCTCTCTTTAATTGACCAAAACCTTCCATTTTTCTCTAAATACAAA<br>GCGATCTGTGTTCTGAGCAACCTTTCCCCGAACACACAGCTTCAGTGCAGCAC<br>GCTGACCTGAGTATCCACCATGTGCCAGGCACAGTGCTGGGCACACGAGGCA<br>CCAAGGTCCGGGCCACCTGCCCGCAGCAAGGCCCAGCTGAGGTGGTGGAGG<br>GAGCCCCTGAGGTCAGGGGCCGTTTCGGTTCAGGGTGGCAGGTGTCCAGCAC<br>TGGGGTATGGCGTCGAGGCTTCCATGGGGTGGGGGAGGCCAGCTTCCTTCTG<br>ACAGGATGGGCGCATACAGTGCCTGGTGTGATTTGTGCACAACCCGTGTTCC<br>AGGTGCACATCC

Mutant-type cDNA	AGGTGTGAGGTGGGAGGATGTGTCCAGCTGGTGGTCTTGGCCCCTCAG<br>GGTGAGAGGACCGTGGGCATGGACGGAAGGGTGGGACGGGAGCCTCA<br>GGGAGACTTGTTATGGGGGCTCCCATTAACTCATACCTGTCTGCAACT<br>ATGAAATCCTAAGAGAACCACAAAATCCATGCCCACAGGGCAAGTAC<br>AGCGGGCAAGGCAGGGGCCTCAGGGCAAGGCCTGACTCATGGCCGTG<br>GCTGACACCTGTCCCGGAGCTGACAGGCTTTGCTCACCTGAAGGCCAT<br>CCCAGAGCTGACAGGCTGTGCTCCGTCTCTCTTCTCCAGGACGGGCAG<br>GTCACCTGCTTCGTGGAAGCTTGCCCCCCTGCCACCTGTGCTGTCCCCG<br>TGAACATCCCAGGGGCCTGCTGTCCAGTCTGCTTACAGAAGAGGGCGG<br>AGGAAAAGCCCTAGGCTCCTGGGAGGCTCCTCAGAGTTTGTCTGCTGT<br>GCCATCGTGAGATCGGGTGGCCGATGGCAGGGAGCTGCGGACTGCAGACCA<br>GGAAACACCCAGAACTCGTGACATTTCATGACAACGTCCAGCTGGTGCTGTT<br>ACAGAAGGCAGTGCAGGAGGCTTCCAACCAGAGCATCTGCGGAGAAGGAGG<br>CACAGCAGGTGCCTGAAGGGAAGCAGGCAGGAGTCCTAGCTTCACGTTAGAC<br>TTCTCAGGTTTTTATTTAATTCTTTTAAAATGAAAAATTGGTGCTACTATTAAA<br>TTGCACAGTTGAATCATTTAGGCGCCTAAATTGATTTTGCCTCCCAACACCAT<br>TTCTTTTTAAATAAAGCAGGATACCTCTATATGTCAGCCTTGCCTTGTTCAGA<br>TGCCAGGAGCCGGCAGACCTGTCACCCGCAGGTGGGGTGAGTCTTGGAGCTG<br>CCAGAGGGGCTCACCGAAATCGGGGTTCCATCACAAGCTATGTTTAAAAAGA<br>AAATTGGTGTTTGGCAAACGGAACAGAACCTTTGATGAGAGCGTTCACAGGG<br>CCACTGTCTGGGGGTGCAGTGCAAGCCCCCGGCCTCTTCCCTGGGAACCTCTG<br>AACTCCTCCTTCCTCTGGGCTCTCTGTAACATTTCACCACACGTCAGCATCTA<br>ATCCCAAGACAAACATTCCCGCTGCTCGAAGCAGCTGTATAGCCTGTGACTCT<br>CCGTGTGTCAGCTCCTTCCACACCTGATTAGAACATTCATAAGCCACATTTAG<br>AAACAGGTTTGCTTTCAGCTGTCACTTGCACACATACTGCCTAGTTGTGAACC<br>AAATGTGAAAAAACCTCCTTCATCCCATTGTGTATCTGATACCTGCCGAGGGC<br>CAAGGGTGTGTGTTGACAACGCCGCTCCCAGCCGGCCCTGGTTGCGTCCACG<br>TCCTGAACAAGAGCCGCTTCCGGATGGCTCTTCCCAAGGGAGGAGGAGCTCA<br>AGTGTCGGGAACTGTCTAACTTCAGGTTGTGTGAGTGCGTTAAAAAAAAAAA<br>AAAAAAAGAATCCCTATACCTCATTTGTATTTTTAAAATGCGTGATGTTTTAT<br>GAAATTGTGTCCATTTTTTAGGTATTAGATATGGCAGAAAAACCATTTCCACT<br>ATGCAAAGTTCTTTTAGACGTCAGTGAAAATCAACTCTCATACCTCATGGTCT<br>CTCTTTAATTGACCAAAACCTTCCATTTTTCTCTAAATACAAAGCGATCTGTG<br>TTCTGAGCAACCTTTCCCCGAACACACAGCTTCAGTGCAGCACGCTGACCTGA<br>GTATCCACCATGTGCCAGGCACAGTGCTGGGCACACGAGGCACCAAGGTCCG<br>GGCCACCTGCCCGCAGCAAGGCCCAGCTGAGGTGGTGGAGGGAGCCCCTGA<br>GGTCAGGGGCCGTTTCGGTTCAGGGTGGCAGGTGTCCAGCACTGGGGTATGG<br>CGTCGAGGCTTCCATGGGGTGGGGGAGGCCAGCTTCCTTCTGACAGGATGGG<br>CGCATACAGTGCCTGGTGTGATTTGTGCACAACCCGTGTTCCAGGTGCACATC<br>C

Yellow denotes the targeted sequence; red indicates the targeted site.

Table S7. Cas9-gRNA sequences used in this study

Name	sequence
h-PXDN gRNA1	GCGTTCACAGGGCCACTGTC
h-PXDN gRNA2	TGCACTGCACCCCCAGACAG
h-PXDN gRNA3	GGGGGTGCAGTGCAAGCCCC

gRNA, guide RNA.

Supplementary Figures

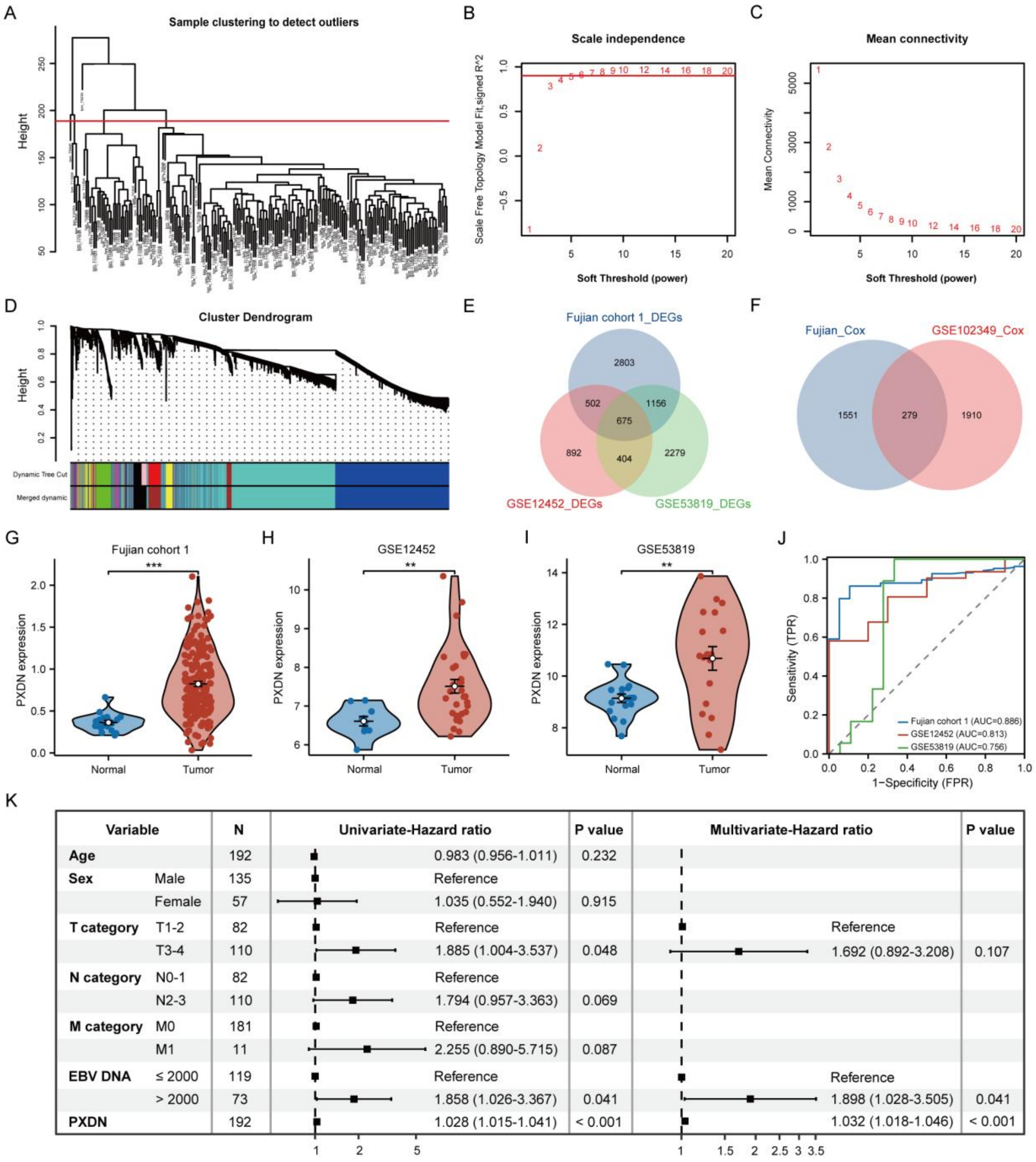

Figure S1. Co-expression network analysis and clinical relevance of PXDN in nasopharyngeal carcinoma. (**A**) Cluster dendrogram of 192 NPC samples (Fujian cohort 1), with two outlier samples. (**B**, **C**) Determination of the soft-thresholding power for scale-free network construction. (**D**) Gene co-expression modules, with distinct colors representing individual modules. (**E**) Venn diagram of differentially expressed genes between NPC and normal nasopharyngeal tissues across three independent cohorts. (**F**) Overlap of DFS-associated prognostic genes in two NPC cohorts. (**G-I**) PXDN expression levels in Fujian cohort 1, GSE12452, and GSE53819 datasets, respectively. (**J**) ROC curves evaluating the diagnostic performance of PXDN in distinguishing NPC from control tissues. (**K**) Univariate and multivariate Cox regression analyses of DFS in Fujian cohort 1. *, P < 0.05, **, P < 0.01, ***, P < 0.001. AUC, area under curve; DEGs, differentially expressed genes; DFS, disease-free survival; EBV DNA, Epstein-Barr virus DNA; FPR, false positive rate; NPC, nasopharyngeal carcinoma; ROC, receiver operating characteristic; TPR, true positive rate.

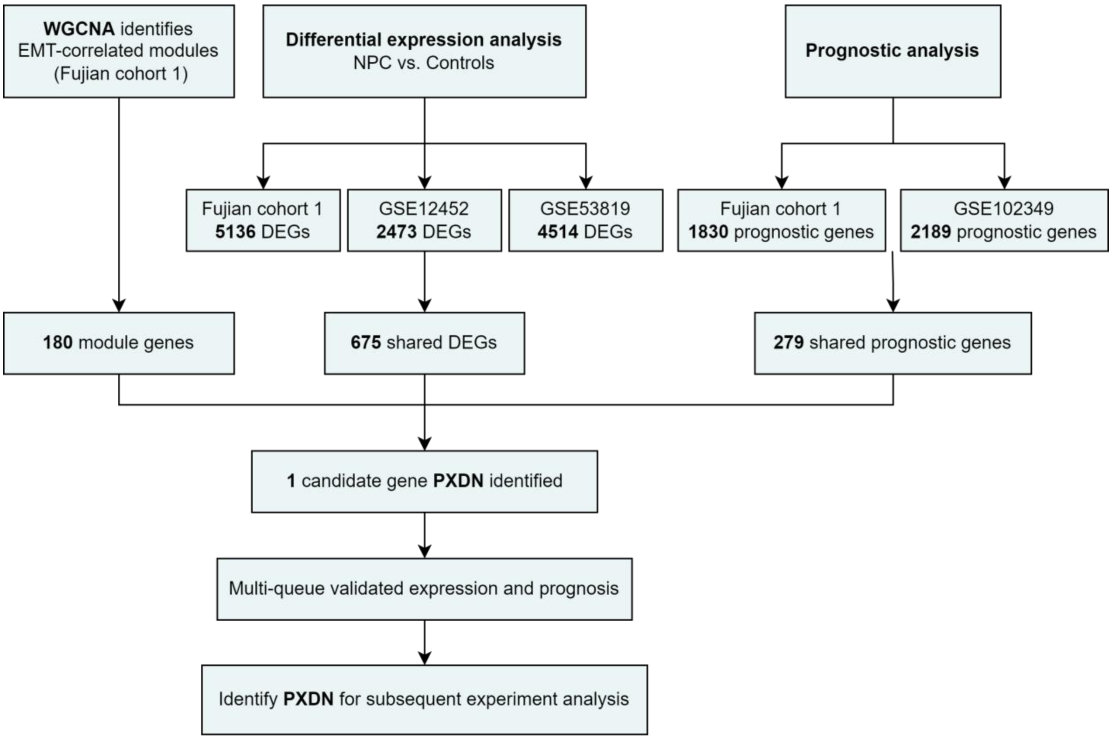

Figure S2. Workflow for identifying PXDN as a key driver of NPC progression. DEGs, differentially expressed genes; EMT, epithelial-mesenchymal transition; NPC, nasopharyngeal carcinoma; WGCNA, weighted gene co-expression network analysis.

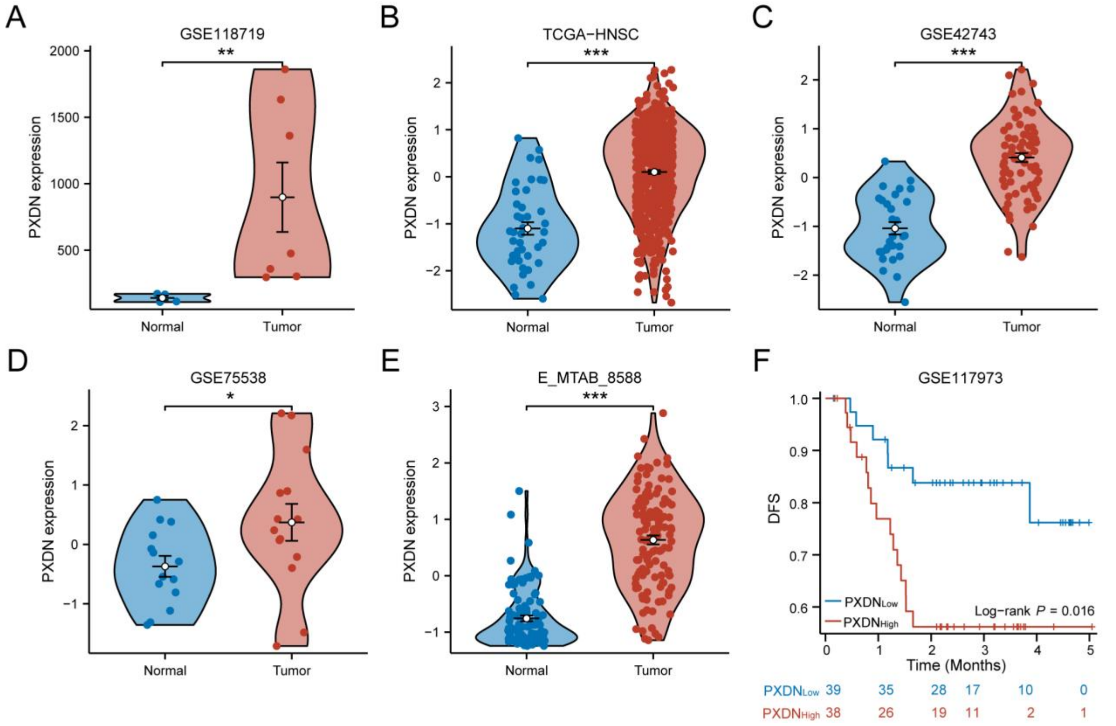

Figure S3. PXDN is overexpressed in head and neck cancer and associated with poor survival. (**A**) PXDN expression in the NPC dataset GSE118719. (**B-E**) PXDN expression in head and neck cancer cohorts: TCGA-HNSC, GSE42743, GSE75538, and E_MTAB_8588. (**F**) Kaplan-Meier curve of DFS in HNSC patients from the GSE117973 cohort, stratified by median PXDN expression. *, P < 0.05, **, P < 0.01, ***, P < 0.001. DFS, disease-free survival; HNSC, head and neck cancer; NPC, nasopharyngeal carcinoma.

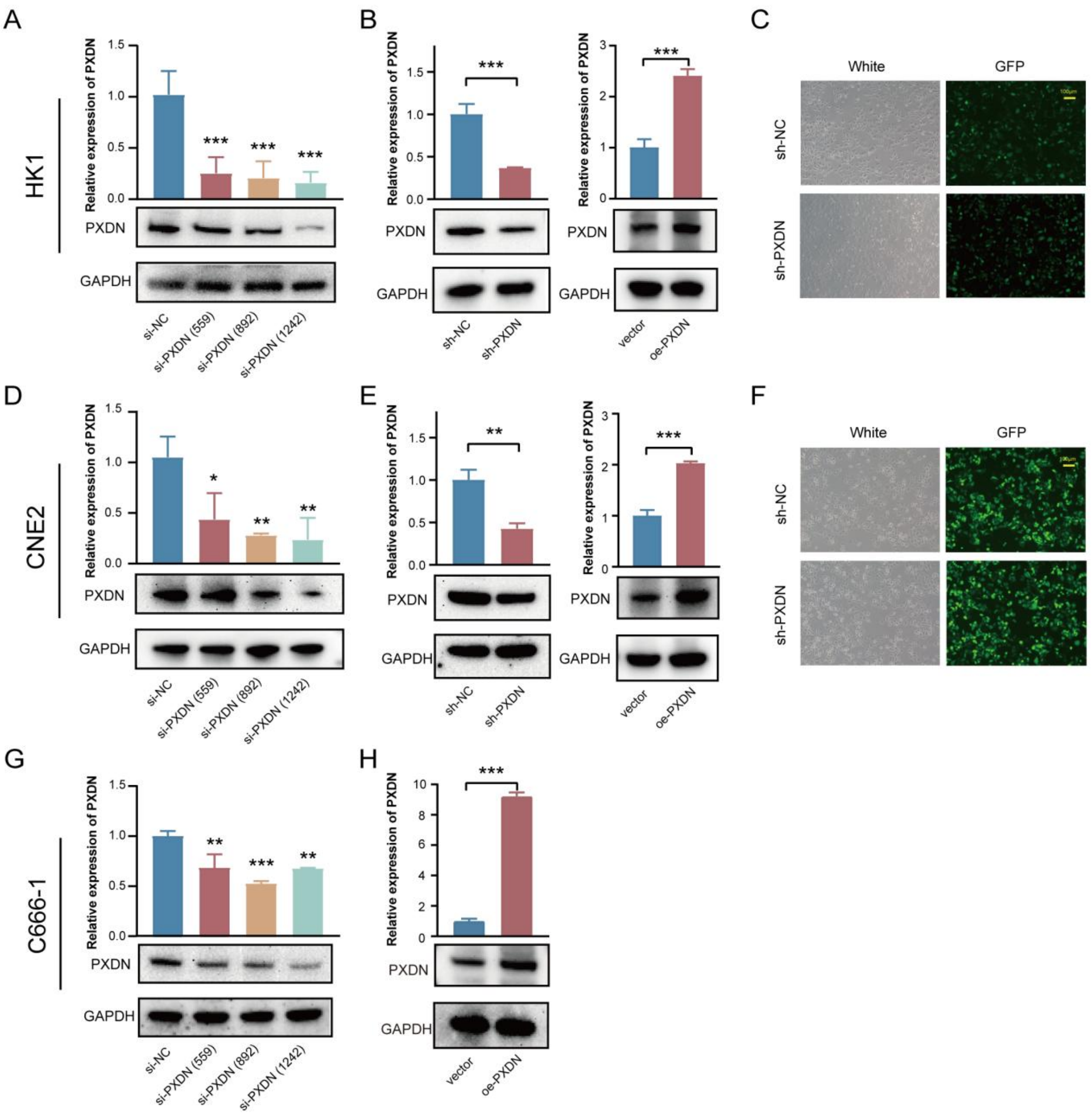

Figure S4. Genetic perturbation of PXDN in NPC cell lines. (**A, D, G**) PXDN knockdown efficiency assessed by qRT-PCR and western blotting following siRNA transfection. (**B, E, H**) PXDN knockdown or overexpression efficiency assessed by qRT-PCR and western blotting in stable sublines generated using lentiviral transduction (selected for optimal knockdown efficiency) in NPC cells. (**C, F**) Fluorescence microscopy images of stable PXDN-knockdown sublines. Scale bar: 100 μm. *, P < 0.05, **, P < 0.01, ***, P < 0.001. GFP, green fluorescent protein; NC, negative control; qRT-PCR, quantitative reverse transcription polymerase chain reaction; siRNA, small interfering RNA.

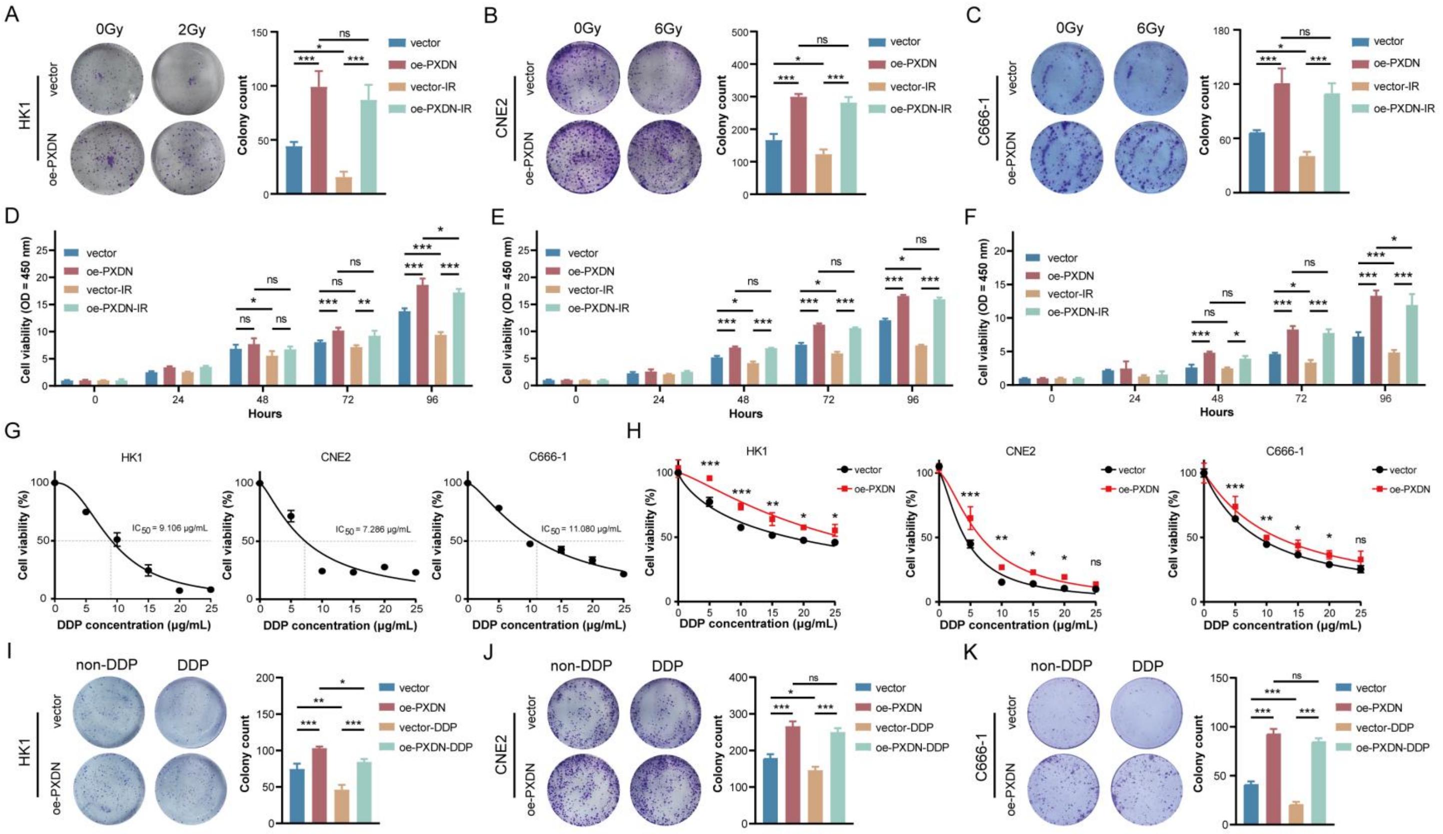

Figure S5. Effects of irradiation and cisplatin on NPC cell proliferation. (**A-C**) Colony formation assays of HK1, CNE2 and C666-1 cells following irradiation exposure. (**D-F**) Cell viability of HK1, CNE2 and C666-1 cells post-irradiation treatment (2 Gy for HK1, 6 Gy for CNE2 and C666-1). (**G**) Cisplatin dose-response curves (0-25 μg/mL, 48 h) for HK1, CNE2 and C666-1 cells. (**H**) Cell viability of HK1, CNE2 and C666-1 cells treated with cisplatin. (**I-K**) Colony formation assays of HK1, CNE2 and C666-1 cells exposed to cisplatin (10 μg/mL for HK1 and C666-1, 7 μg/mL for CNE2). *, $P < 0.05$, **, $P < 0.01$, ***, $P < 0.001$. DDP, cisplatin; IR, irradiation.

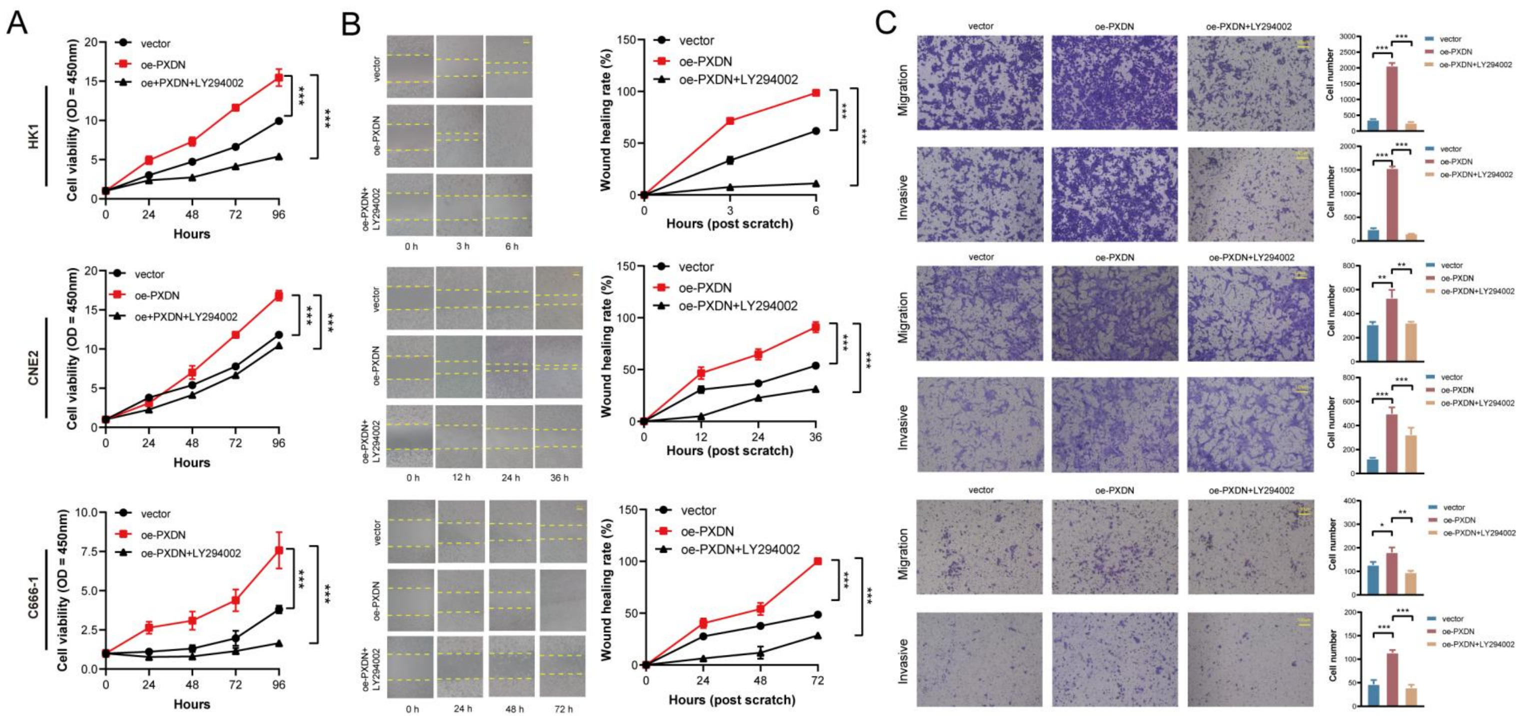

Figure S6. Effects of the PI3K inhibitor LY294002 on PXDN-overexpressing NPC cells. (**A**) Proliferation of NPC cells (vector control, oe-PXDN, and oe-PXDN + LY294002) measured by CCK-8 assay over 96 h. (**B**) Representative images (left) and quantification (right) of wound-healing migration assays. Scale bar: 100 μm. (**C**) Representative images (left) and quantification (right) of Transwell migration and Matrigel invasion assays. Scale bar: 100 μm. *, P < 0.05, **, P < 0.01, ***, P < 0.001. CCK-8, cell counting kit-8.

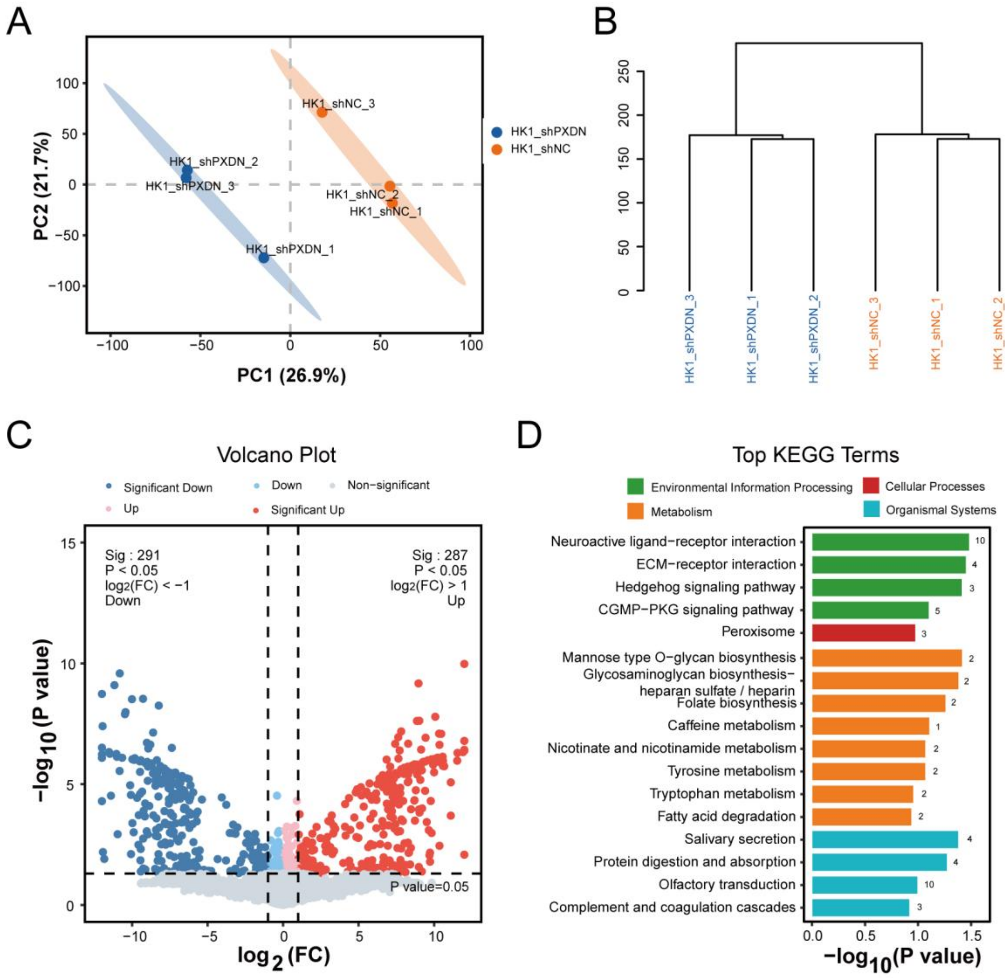

Figure S7. Proteomic profiling of HK1 cells with PXDN knockdown. (**A**, **B**) Unsupervised analysis of proteomic data showing principal component analysis and hierarchical clustering of samples. (**C**, **D**) Differentially expressed proteins between PXDN-knockdown and control cells, with the top enriched KEGG pathways. ECM, Extracellular matrix; FC, fold change; KEGG, Kyoto Encyclopedia of Genes and Genomes; PC, principal component.

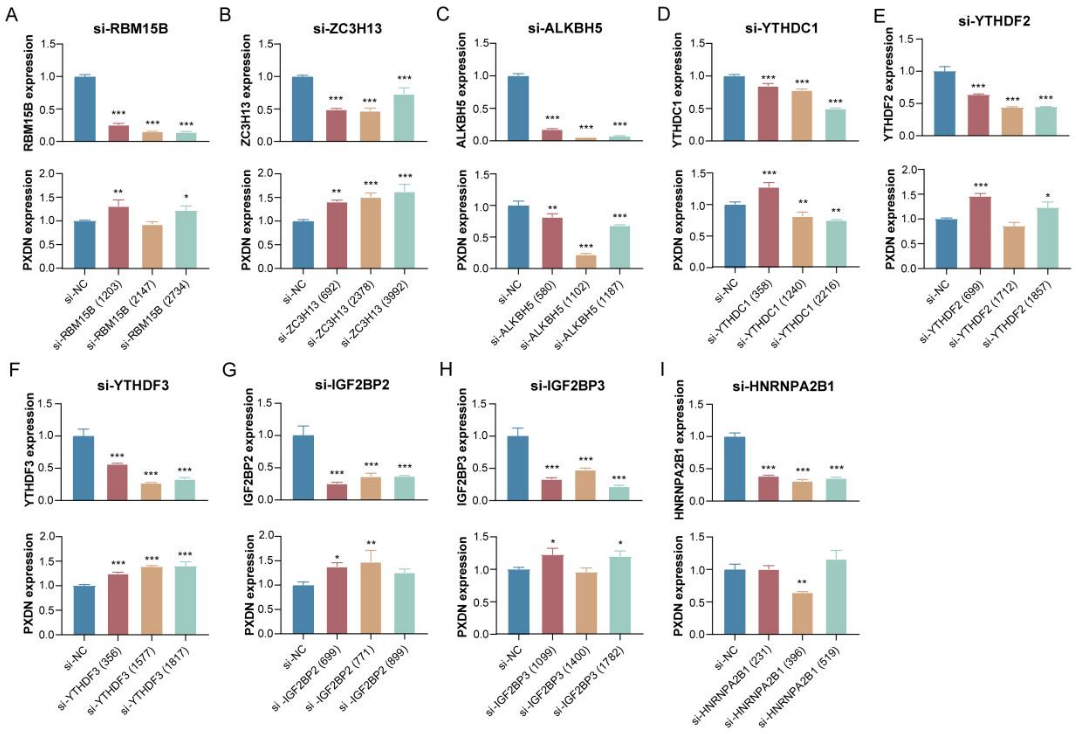

Figure S8. The relative expression levels of m^6A regulators and PXDN in knockdown sublines assessed by qRT-PCR. The knockdown sublines of m^6A regulators, included (A) RBM15B, (B) ZC3H13, (C) ALKBH5, (D) YTHDC1, (E) YTHDF2, (F) YTHDF3, (G) IGF2BP2, (H) IGF2BP3, and (I) HNRNPA2B1. m^6A, N6-methyladenosine; NC, negative control; qRT-PCR, quantitative reverse transcription polymerase chain reaction.

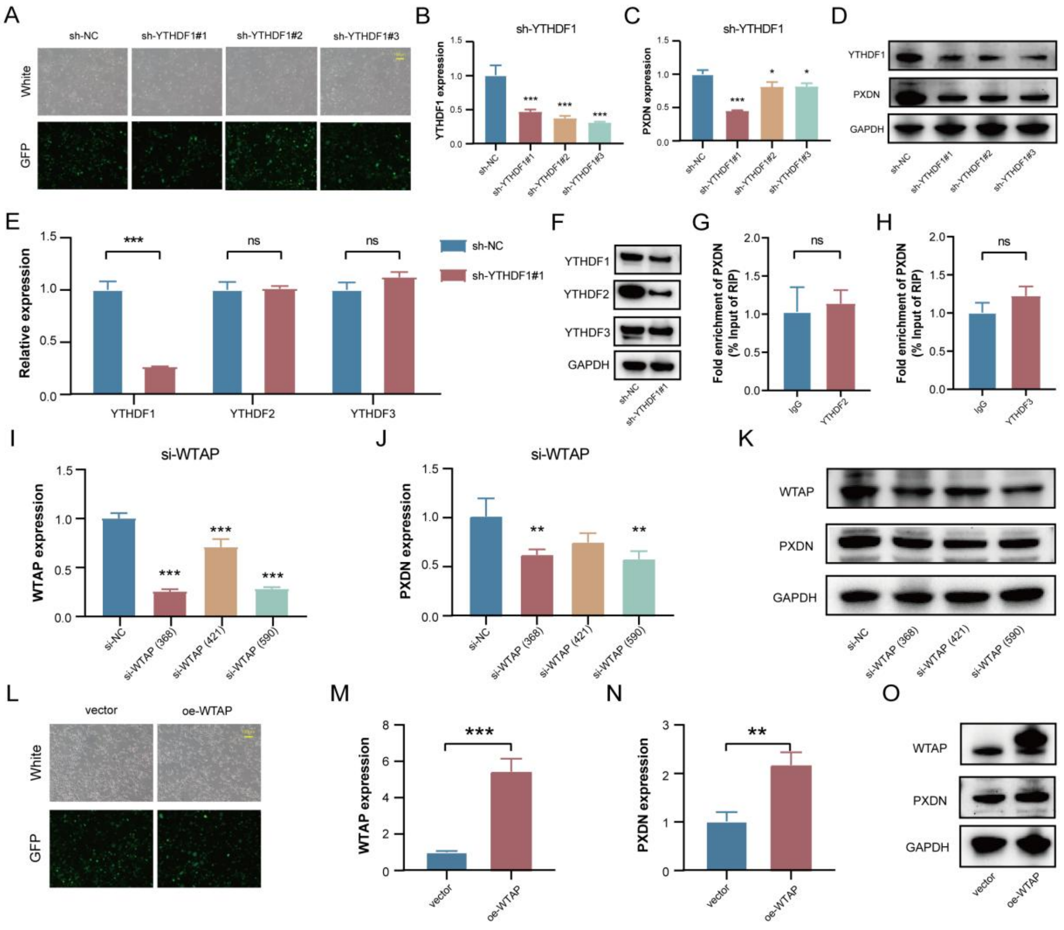

Figure S9. YTHDF1 and WTAP-mediated regulation in NPC cells. (**A**) Fluorescence microscopy images of stable YTHDF1-knockdown sublines. (**B-D**) Relative expression of YTHDF1 and PXDN in knockdown sublines assessed by qRT-PCR and western blotting. (**E**, **F**) Compensatory expression of YTHDF2 and YTHDF3 in YTHDF1-knockdown cells analyzed by qRT-PCR and western blotting. (**G**, **H**) RIP assays showing absence of PXDN binding to YTHDF2 or YTHDF3 in HK1 cells (qRT-PCR analysis). (**I-K**) Relative expression of WTAP and PXDN in WTAP knockdown assessed by qRT-PCR and western blotting following siRNA transfection. (**L**) Fluorescence microscopy images of stable WTAP-overexpressing sublines. (**M-O**) Relative expression of WTAP and PXDN in overexpressing sublines assessed by qRT-PCR and western blotting. Scale bar: 100 μm. *, $P < 0.05$, **, $P < 0.01$, ***, $P < 0.001$. GFP, green fluorescent protein; NC, negative control; qRT-PCR, quantitative reverse transcription polymerase chain reaction; RIP, RNA immunoprecipitation; siRNA, small interfering RNA.
